# Gut microbiota and resistome profiles of Swiss expatriates in Africa revealed by Nanopore metagenomics

**DOI:** 10.1038/s41598-026-38302-3

**Published:** 2026-02-03

**Authors:** Edgar I. Campos-Madueno, Claudia Aldeia, Andrea Endimiani

**Affiliations:** Institute for Infectious Diseases (IFIK), University of Bern, Friedbühlstrasse 25, 3001 Bern, Switzerland

**Keywords:** Shotgun, Europe, Stool, ARGs, Plasmids, Expats, Genetics, Microbiology

## Abstract

The gut microbiota and resistome may change upon exposure to environments with high prevalence of multidrug-resistant pathogens, potentially impacting health and contributing to the spread of antimicrobial resistance genes (ARGs). In this context, expatriates may acquire endemic microbial communities and ARGs while living abroad. In this work, we investigated the microbiota and resistome of Swiss expatriates living in African countries using Nanopore shotgun metagenomics (SMS).

Stool samples from expatriates residing in African and European countries (*n* = 33 and *n* = 39, respectively) were sequenced using Nanopore V14 chemistry. Taxonomic and resistome profiling was performed with Kraken2 and ResFinder, respectively. Diversity metrics (e.g., Shannon, Simpson) assessed microbial composition. ARG and bacteria associations were determined using GTDB-Tk on metagenome-assembled genomes (MAGs). Plasmid-borne ARGs were characterized with PlasmidFinder.

Our results indicated that microbiota composition did not differ between expatriates in African and European countries. However, resistome analysis revealed a higher prevalence of tetracycline (*tet*) and folate pathway antagonist (*dfr*, *sul*) ARGs in those residing in Africa, suggesting adaptation to the local microbial environment or antibiotic policy. Unique plasmid families were also identified in Gram-negative (IncF) and -positive (repUS43) bacteria across African and European cohorts, indicating the potential for ARG dissemination *via* mobile genetic elements. Overall, Nanopore-based SMS may provide an alternative approach to monitor microbiota and resistome dynamics, and thus assisting early epidemiological surveys.

**Supplementary Information:**

The online version contains supplementary material available at 10.1038/s41598-026-38302-3.

## Introduction

There is growing evidence that exposure to regions endemic for multidrug-resistant (MDR) bacteria can influence the human gut microbiota composition and its associated resistome^1–5^. In this context, healthy people living abroad (e.g., expatriates) in countries located in the African continent may acquire and further disseminate antimicrobial resistance genes (ARGs). Therefore, understanding this complex dynamic is essential for public health initiatives and global efforts against antimicrobial resistance (AMR).

Several surveys, particularly those involving international travelers to African countries (e.g., Tanzania), have provided strong evidence that healthy individuals can return home colonized in the gut with MDR Enterobacterales (Ent) (e.g.,^6–14^). Such Ent are often associated with extended-spectrum β-lactamases (ESBLs) of the CTX-M type, and may also harbor the *mcr-1* gene, conferring resistance to colistin, a last-resort antibiotic^12–14^.

Recently, we demonstrated that Swiss expatriates, can also return colonized at gut level with epidemiologically significant MDR-Ent after living long-term in African countries^15^. However, most surveys to date have concentrated their efforts on intestinal colonization typically defined by the carriage of at least a single bacterium of a desired phenotype (e.g., MDR-Ent grown on selective media)^16^. While epidemiologically important, this approach limits our understanding of how exposure to high-endemic regions may influence the broader gut microbial community and its associated resistome.

To develop a more complete view, there is growing interest in complementing traditional culture-based methods and strain whole-genome sequencing (WGS) with culture-independent approaches^17,18^. While strain WGS offers insightful details, its use does not describe the diversity of microbial taxa and ARGs present in the human gut. Shotgun metagenomic sequencing (SMS), traditionally based on Illumina short-reads, enables comprehensive profiling of both the taxonomic composition and the resistome, unlike 16S rRNA-amplicon sequencing, which is limited to taxonomic characterization^19^. Importantly, SMS can detect clinically important ARGs and identify their associated mobile genetic elements (MGEs), such as the plasmids that facilitate their spread, especially when a long-read (e.g., Nanopore) or hybrid (e.g., both Illumina and Nanopore) approach is implemented (e.g.,^17,20–24^).

As AMR continues to place a heavy burden in lower income settings, including the African countries^25^, such approaches hold great promise. Emerging metagenomic studies in African populations have already begun to uncover distinctive microbiota and resistome signatures, offering new insights into the dynamics of AMR reservoirs^23,26^. However, to better understand the global implications of these findings, more comparative metagenome studies (e.g.,^1–5^) between low and high endemic regions (e.g., European vs. African populations), are urgently needed.

In this study, we implemented a Nanopore-SMS approach to assess distinct differences in microbiota composition - in particular resistome profiles (i.e., ARGs) and their associated plasmids inferred from meta-assembled genomes (MAGs). We provided new insights into how long-term living in high-risk regions, such as African countries^15^, may influence broader resistome changes beyond intestinal colonization by MDR-Ent in Swiss expats. This highlights the potential of a Nanopore-SMS approach as a powerful tool for exploratory and comprehensive epidemiological surveys.

## Results

### Characteristics of the stool samples selected for this study

A total of 72 stool samples included in this study were derived from a previous prospective prevalence study on intestinal colonization of Swiss expats^15^. Of them, 33 (45.8%) were from Swiss expats living in Europe, and 39 (54.2%) from Swiss expats living in African countries. Four of the 33 European stool samples (12.1%) and 19 of the 39 African stool samples (48.7%) were culture-positive for intestinal colonization with CTX-M-producing *E. coli* (Table S1). Moreover, a prior risk factor analysis identified continent of residence as the only variable significantly associated with increased odds of colonization with MDR-Ent (continent: Africa; adjusted odds ratio = 3.4, 95% confidence interval 1.0–11.0), compared to expatriates residing in Europe (Table S1)^15^.

To extend these findings, here we investigated whether changes in gut microbiota composition and resistome profiles differed by intestinal colonization status and continent. For simplicity, stool samples from Swiss expats who lived in European and African countries are hereafter referred to as European and African stools, respectively.

### Nanopore metagenomic sequencing (Nanopore-SMS)

The 72 stool samples were subjected to Nanopore-SMS in duplicate (*n* = 144 total) and randomized across 7 different sequencing batches. Together, all sequencing runs generated a mean of 409,143.3 reads, with a mean of 1791.6-bp read length (median: 806-bp), 12.7 quality score (Q-Score), and 3921.3-bp read length N50. After read preprocessing (i.e., barcode trimming and host decontamination), the overall mean sequencing depth corresponded to 386,328 reads (~ 5.6% read loss), 1738.7-bp read length (median: 781-bp), 16.7 Q-Score and 4076.5-bp read length N50 (Fig. S1; Source Data file). Notably, across all sequencing output metrics discussed above, no statistically significant differences were observed between European and African stools. Therefore, to enhance sequencing depth and improve the resolution of microbiota and resistome profiling, duplicate samples were concatenated prior to downstream analyses. Moreover, to provide a reliable overview of microbiota composition across all samples, community analyses were performed at the genus level, while MAGs were reserved specifically for ARG screening, plasmid identification, and high-resolution taxonomic classification using GTDB-Tk. These MAG-derived results were subsequently contextualized with the read-based findings.

### Microbiota profiles of European and African stools

Kraken2 classified reads at the genus level resulted in 1,831 genera after preprocessing. To account for differences in sequencing depth (e.g., S1-GRB-03), read counts were normalized by subsampling (i.e., rarefying without replacement) (Fig. S2a). After normalization, 1765 genera remained (194,342 read counts per sample), indicating that 66 global genera were removed from the dataset due to having zero counts across all samples. Moreover, a mean of 105 genera (range: 0-268) was lost per sample, reflecting a reduction in observed richness (Fig. S2b). This reduction mostly involved rare genera and was therefore deemed acceptable for further analyses.

**Fig. 1:**
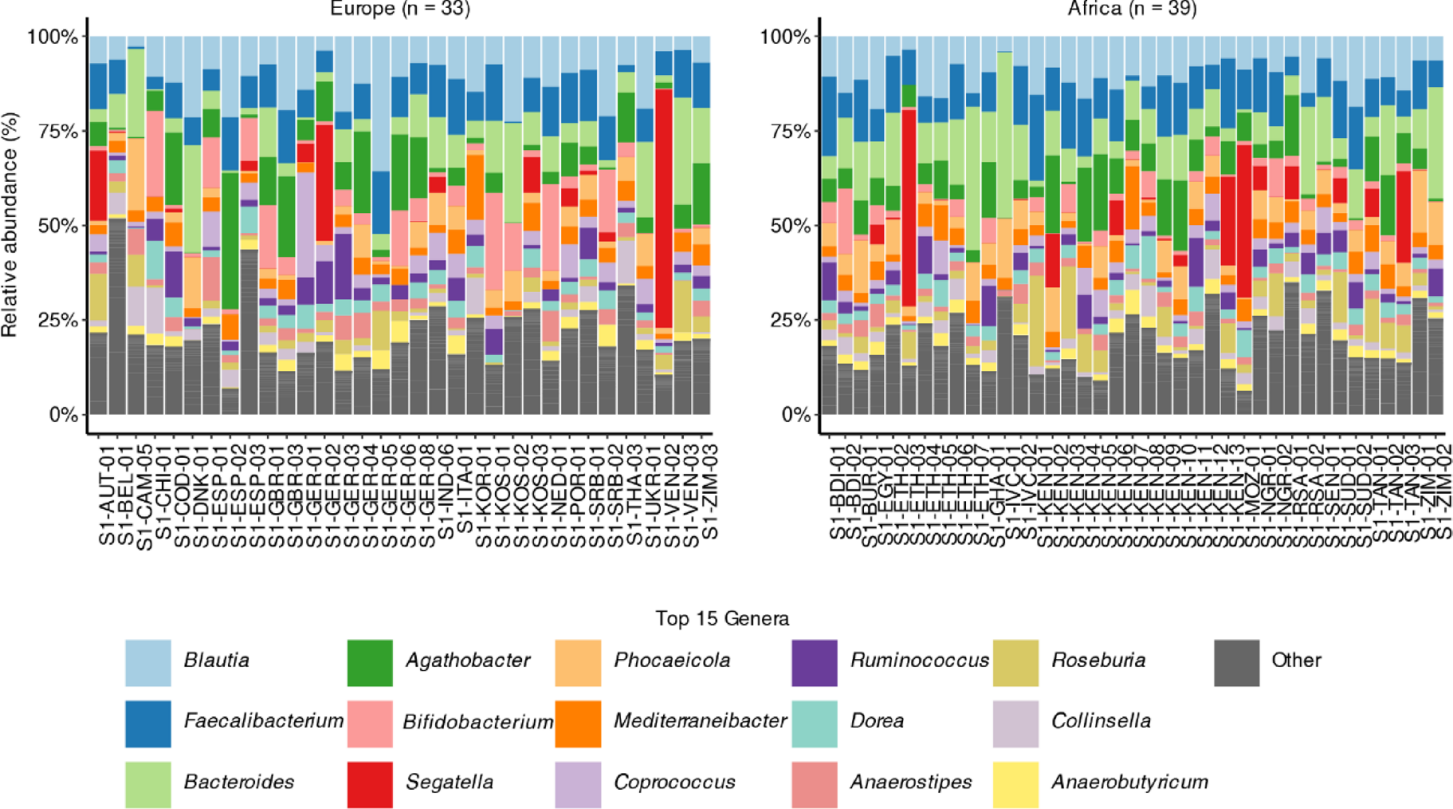
Relative abundance of bacteria at the genus level in European (*n* = 33) and African (*n* = 39) stool samples. The top 15 genera found in both European and African samples are shown in different colors, while the remaining genera are shown as ‘other’ and colored in grey. Read counts were normalized by subsampling and relative abundance. Source data are provided as a Source Data file.

**Fig. 2:**
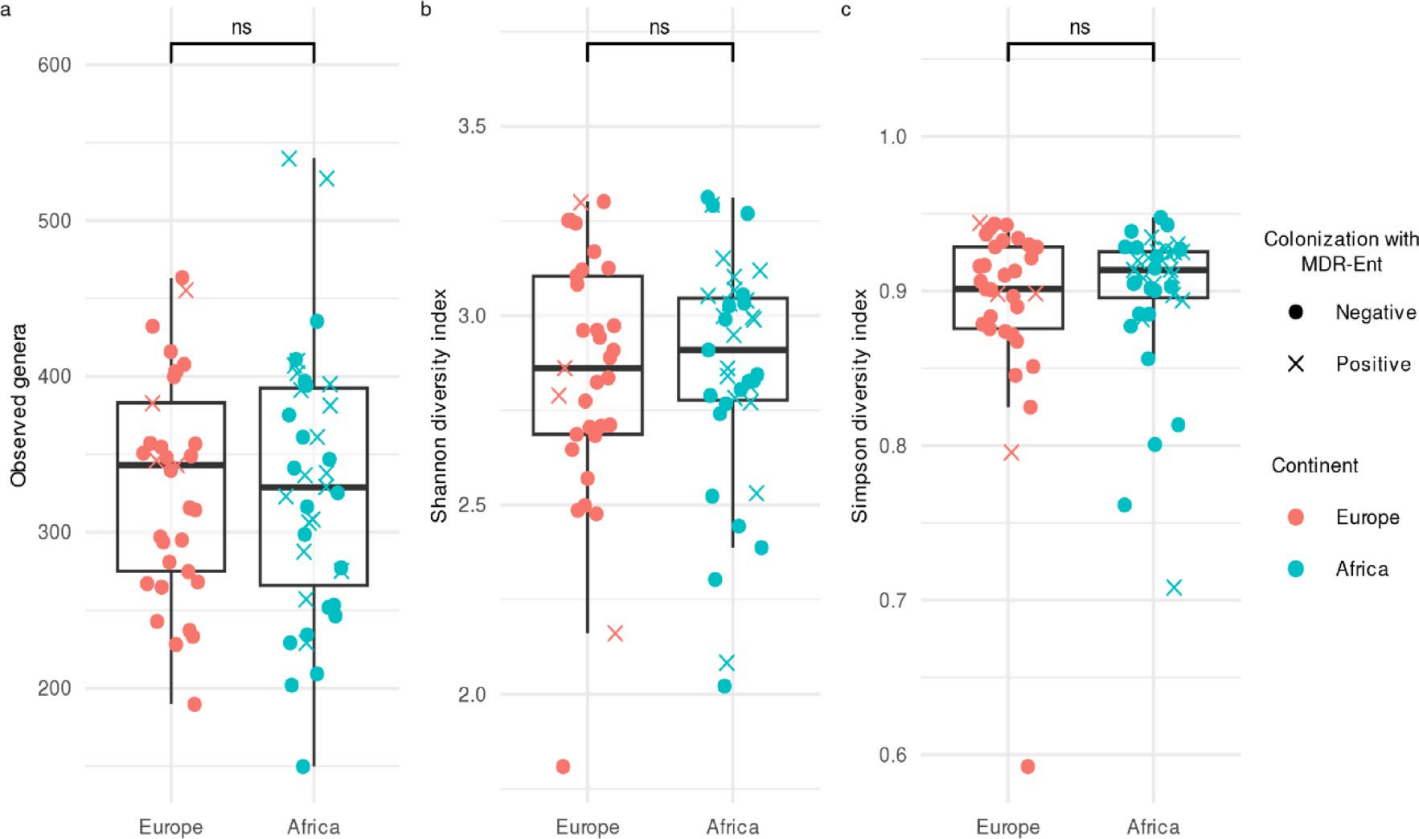
Alpha diversity indices stratified by European (*n* = 33) and African (*n* = 39) stool samples using the subsampled dataset. Boxplots (**a**, **b**) and (**c**) show the observed genera, Shannon and Simpson diversity indices, respectively. Circles are colored by continent, while intestinal colonization status with MDR-Ent is represented by a circle or an ‘X’ for negative or positive, respectively. In all boxplots, the line in the center of the box represents the median, while the 25th and 75th percentiles are the lower and upper bounds of the box, respectively. Lower and upper whiskers extend the box, representing data points outside the interquartile range (1.5 times). Statistical significance between groups (Europe vs. Africa) is indicated by the significance level marked above the bracket (ns, not significant). The unpaired non-parametric Wilcoxon Rank Sum test (two-sided) was used to compare groups in boxplot (**b**, **c**), while an unpaired Student’s t-test (two-sided) was used to compare groups in boxplot (**a**). The exact *p*-values for boxplots (**a**, **b**, and **c**) are 0.96, 0.69, and 0.56, respectively. Source data are provided as a Source Data file.

Relative abundance analysis of bacterial genera revealed shared commensal taxa from the Clostridia (e.g., *Blautia*, *Faecalibacterium*) and Bacteroidia (e.g., *Bacteroides*, *Prevotella*) classes in both European and African stools (Fig. 1). Despite a slight difference in the relative abundance of the genus *Bacteroides* between African and European stools (median: 10.1% vs. 5.4%), no significant differences in overall microbial composition were observed between the two groups (Fig. 1; Source Data). This was supported by within-sample (alpha) diversity metrics, which showed no statistically significant differences in observed genera counts (*p* = 0.96), Shannon diversity (*p* = 0.69), or Simpson (*p* = 0.56) indices, despite a higher number of colonization-positive samples among African stools (*n* = 19/39 vs. *n* = 4/33 for European samples) (Fig. 2). Between-sample (beta) diversity analysis using Bray-Curtis distances also indicated no significant difference in community dispersion between European and African stools (*p* = 0.073) (Fig. S3a). Similarly, non-metric multidimensional scaling (NMDS) revealed no clear clustering patterns distinguishing the two groups (Fig. S3b). This observation was supported by a permutational multivariate analysis of variance (PERMANOVA) test, which revealed that continent explained ~ 2% of the variance in Bray-Curtis dissimilarities between samples (R^2^ = 0.02, F = 1.39, *p* = 0.17), indicating no statistically significant clustering by continent.

### Resistomes associated to European and African stools

**Fig. 3:**
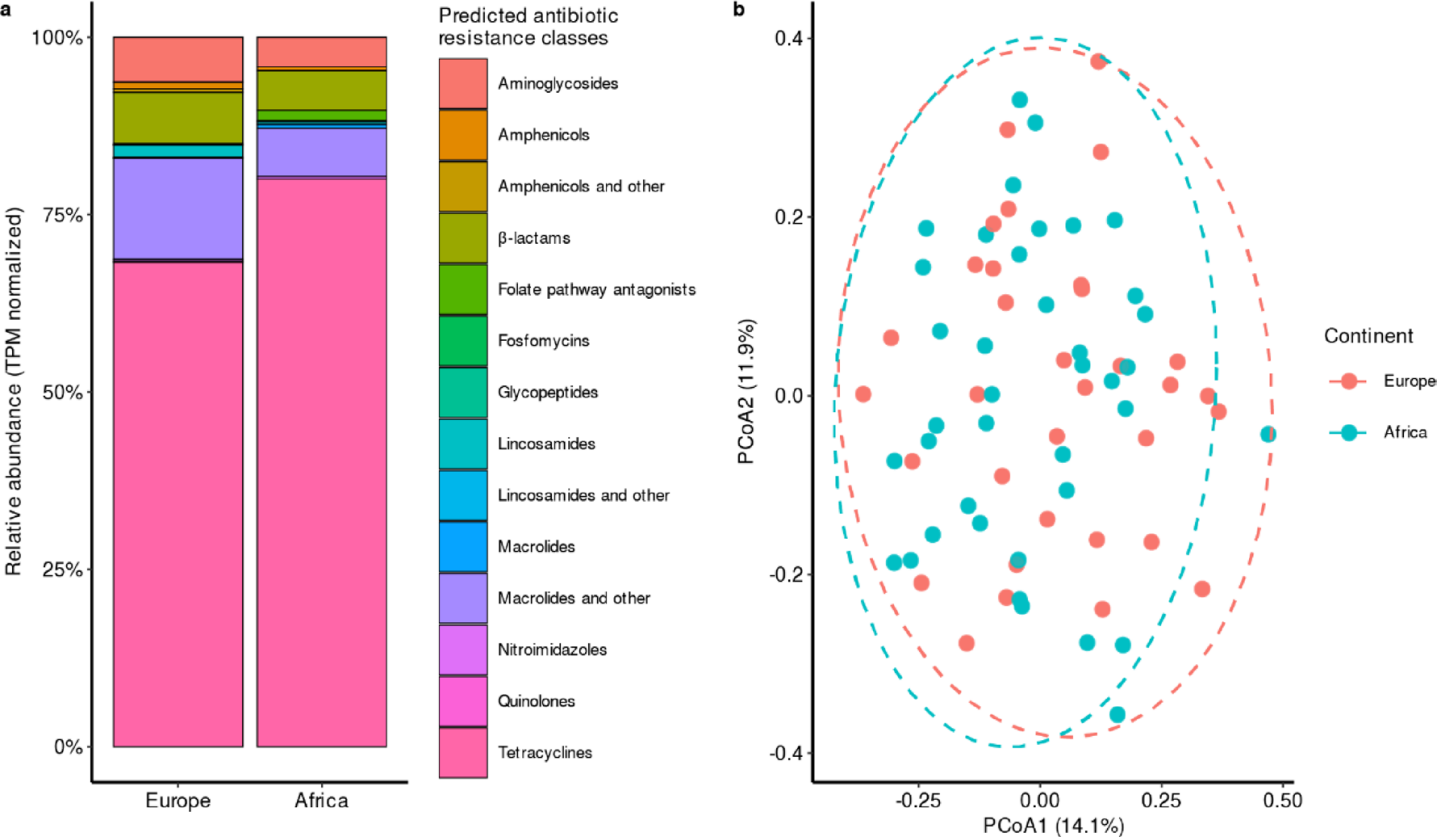
Relative abundance of predicted antibiotic resistance classes and dimensionality reduction of antimicrobial resistance gene (ARG) profiles in European (*n* = 33) and African (*n* = 39) stool samples. Plot (**a**) shows transcripts per million (TPM)-normalized and relative abundance-transformed ARG counts (overall, *n* = 134 ARGs), grouped by predicted antibiotic resistance class and stratified by continent. Plot (**b**) represents the Principal Coordinates Analysis (PCoA) based on Bray–Curtis distances of TPM-normalized ARGs, comparing European and African stool samples. The percentage of variance explained by each axis is indicated in the plot. Dashed 95% confidence ellipses represent the distribution of samples per continent. See Supplementary Data 1 for definitions of ARGs and the corresponding antibiotic resistance classes. Source data are provided as a Source Data file.

**Fig. 4:**
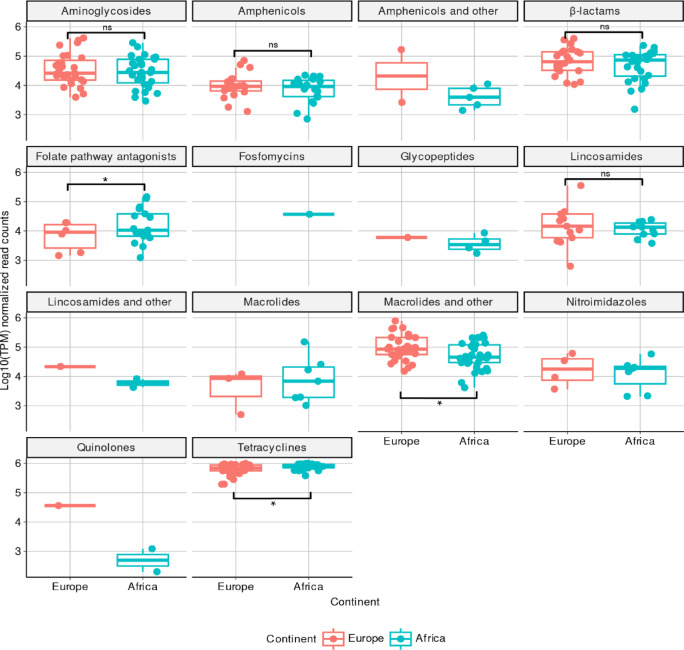
Relative abundance distribution of TPM-normalized antibiotic resistance gene (ARG) counts in European and African samples by ARG class. For visualization, zero read counts were excluded, and values were log10-transformed. Each data point is represented by a circle, colored by continent. In all boxplots, the center line indicates the median; the box bounds represent the 25th and 75th percentiles. Whiskers extend to 1.5 times the interquartile range. Statistical comparisons between continents (Europe vs. Africa) are indicated by brackets, with significance levels denoted as follows: ns (not significant), * (*p* < 0.05). Group comparisons were performed using a two-sided, unpaired exact Wilcoxon-Mann-Whitney test. Statistical valid comparisons were only done when both groups (Europe and Africa) contained each at least 5 observations (data points). The exact *p*-values for the aminoglycosides, amphenicols, β-lactams, folate pathway antagonists, lincosamides, macrolides and other, and tetracyclines boxplots are 0.62, 0.27, 0.96, 0.01, 0.25, 0.02, and 0.03, respectively. Source data are provided as a Source Data file.

A total of 134 ARGs normalized to transcripts per million (TPM) were detected in European (*n* = 93 ARGs) and African (*n* = 113 ARGs) stools, which belonged to 14 antibiotic resistance classes (Fig. 3a; Supplementary Data 1). Considering all ARGs within each antibiotic resistance class between European and African stools, a PERMANOVA test showed that ARG abundance profiles did not significantly differed by continent (R^2^= 0.02, *p* = 0.29) (Fig. 3b). Nevertheless, African stools contained a statistically significantly larger proportion of ARGs with predicted resistance to folate pathway antagonists (*p* = 0.01) and tetracyclines (*p* = 0.03), whereas European stools only for macrolides and others (*p* = 0.02) (Figs. 3a and 4).

We observed differences in the unique ARGs associated with European and African stool samples (Table 1). For example, a greater number of unique tetracycline resistance genes (*tet*) were identified in African stools compared to European ones (*n* = 8/40 vs. *n* = 3/40, respectively). Additionally, ARGs predicted to encode folate pathway antagonists, such as *dfr* and *sul2*, were more unique to African samples (6/9 vs. 0/9, respectively). However, when considering all ARG classes, the number of unique ARGs per sample was not statistically associated with either continent group (Table 1; Fisher’s exact test, *p*-value range = 0.2 to 1; adjusted *p*-value = 1).

**Table 1:** Differences in unique antimicrobial resistance genes (ARGs) per antimicrobial resistance class between European and African stools.

ARG class	Unique ARGs^a^		Shared ARGs^b^	Total ARGs ^c^	*p*-value ^d^	*p*-value (adj) ^e^	Unique ARGs ^f^	
	Europe	Africa					Europe	Africa
Tetracyclines	3	8	29	40	0.2	1	*tetA(46)*_1_HQ652506, *tet(M)*_8_X04388, *tet(X6)*_1_MN507533	*tet(O/32/O)*_6_NG_048124, *tet(O/32/O)*_2_AJ295238, *tet(O/32/O)*_5_FP929050, *tet(M)*_2_X90939, *tet(M)*_10_EU182585, *tet(W)*_2_AY049983, *tetA(P)*_2_L20800, tet(M)_1_X92947
Macrolides and other	3	3	13	19	1	1	*msr(C)*_2_AF313494, *erm(X)*_1_M36726, *msr(C)*_1_AY004350	*erm(G)*_2_L42817, *erm(B)*_21_U35228, *erm(T)*_2_AY894138
Aminoglycosides	7	7	8	22	1	1	*aph(2)-Ia*_2_AP009486, *aph(2)-Ia*_3_AJ536195, *aac(6)-Ii*_1_L12710, *npmA*_1_AB261016, *aadA8b*_2_AM040708, *aadA2*_2_JQ364967, *aadA1*_4_JQ480156	*aadA1*_3_JQ414041, *aadA1*_5_JX185132, *ant(6)-Ia*_2_KF421157, *aac(6)-Iid*_1_AJ584700, *aph(2)-Ig*_1_CP004067, *aph(6)-Id*_4_CP000971, *aadA5*_1_AF137361
Lincosamides	0	1	2	3	1	1	None	*lnu(B)*_2_JQ861959
Quinolones	1	2	0	3	1	1	*qnrS1*_1_AB187515	*qepA4*_1_KX580704, *qnrB19*_1_EU432277
Lincosamides and other	1	1	0	2	1	1	*lsa(A)*_1_AY225127	*lsa(E)*_1_JX560992
β-lactams	3	5	8	16	0.7	1	*bla*_TEM−30__1_AJ437107, *bla*_TEM−95__1_AJ308558, *bla*_TEM−1D__1_AF188200	*bla*_OXA−1__1_HQ170510, *bla*_ACT−6__1_FJ237366, *cepA*_1_L13472, *cepA*_1_U05887, *cfiA14*_1_FM200789
Folate pathway antagonists	0	6	3	9	0.2	1	None	*dfrA7*_1_AB161450, *sul2*_3_HQ840942, *dfrA17*_1_FJ460238, *sul2*_18_AJ830714, *dfrA14*_4_AF393510, *sul2*_6_FN995456
Amphenicols	1	2	3	6	1	1	*floR*_2_AF118107	*catA1*_1_V00622, *Cfr(E)*_1_NG_070225
Nitroimidazoles	0	1	2	3	1	1	None	*nimD*_1_X76949
Macrolides	2	1	1	4	1	1	*mef(A)*_1_AJ971089, *ere(D)*_1_KP265721	*mef(A)*_2_U83667
Amphenicols and other	0	0	2	2	1	1	None	None
Glycopeptides	0	3	1	4	0.4	1	None	*VanHDX*_4_AY082011, *VanHDX*_5_AY489045, *VanG2XY*_1_FJ872410

^a^Number of unique ARGs found in both European and African samples.

^b^Shared ARGs between both European and African samples.

^c^Total count of all ARGs.

^d^The *p*-value of the Fisher exact test result assessing the significance of unique ARGs between European and African samples.

^e^The *p*-value adjusted for multiple testing (FDR corrected).

^f^The list of ARG hits from the ResFinder database unique to European and African samples. See Supplementary Data 1 and Supplementary Data 3 for ARGs definition and the complete ARG dataset, respectively.

In both Europe and Africa, a large proportion of MAGs associated with ARGs were classified within genomic sequences corresponding to the order Eubacteriales (formerly Clostridiales), based on CheckM marker lineages [e.g., o__ClostridialesUID1212]. Specifically, Eubacteriales-associated MAGs accounted for 46.5% and 46.8% of ARG-linked MAGs in European and African stools, respectively (Table S2). This pattern was further supported by read-based taxonomic classification (see section above), which revealed that 10 of the top 15 genera (e.g., *Blautia*, *Faecalibacterium*,* Agathobacter*), belonged to the class Clostridia (Fig. 1). Moreover, a high-resolution taxonomic classification with GTDB-Tk of MAGs associated with ARGs showed that the genera *Ruminococcoides* and *Bifidobacterium* in Europe, and *Ruminococcoides* and *Bacteroides* in Africa, were often associated with more tetracycline- (*tet*) and macrolide-associated ARGs (*erm*), respectively (Table S3; Supplementary Data 2). Lastly, while the *Escherichia* genus was less prevalent than *Ruminococcoides*, *Bifidobacterium*, and *Bacteroides*, it was found associated with clinically-relevant ARGs such as *bla*_TEMs_ and *bla*_CTX−M−15_, identified in *E. coli* from European and African stools, respectively (Table S3; Supplementary Data 2).

### Characteristics of plasmids associated with ARGs

**Fig. 5:**
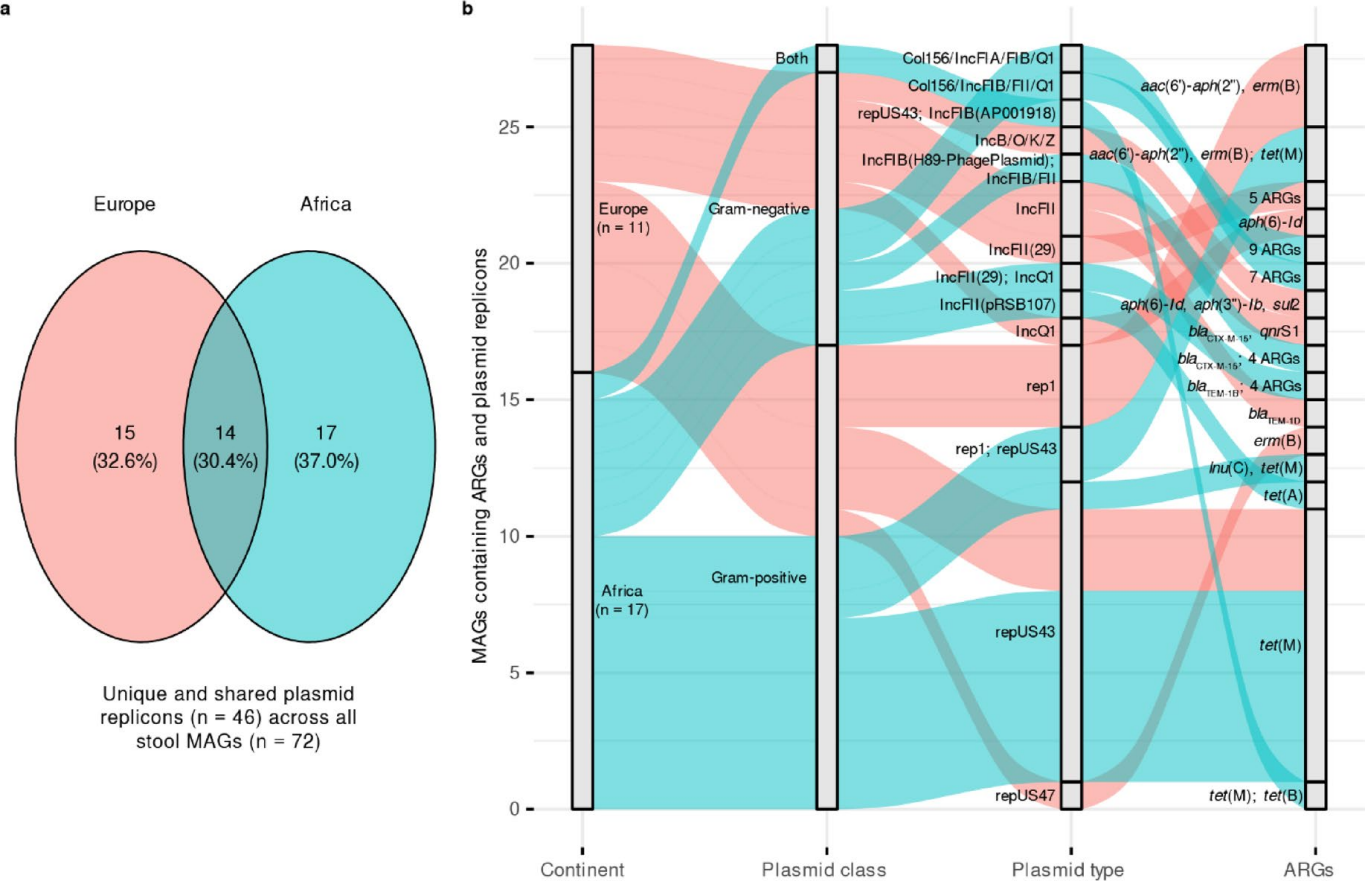
Plasmid replicons associated with antimicrobial resistance genes (ARGs) in all stool MAGs (*n* = 72). The Venn diagram (**a**) illustrates the number of unique and shared plasmid replicon sequences among all stool metagenome-assembled genomes (MAGs) in European and African stool samples. The alluvial diagram (**b**) shows the flow of shared data among continent, plasmid class, plasmid type, and ARGs. The number of MAGs with plasmid replicon sequences from European and African stool samples is shown in the ‘Continent’ column. More than one plasmid replicon type (noted as ‘Both’ under ‘Plasmid class’) is separated by a semicolon under ‘Plasmid type.’ ARGs corresponding to a specific replicon-type plasmid are also separated by a semicolon and follow the same order. For visualization purposes, the plot only shows ARGs associated with plasmid replicon sequences within MAG contigs. The complete dataset is provided as a Source Data file.

A total of 46 plasmid replicon sequences (i.e., putative plasmids) were identified across the 72 MAGs from European and African stools. Of these, 15 and 17 plasmid replicon sequences were unique to European and African stools, respectively, while 14 were shared (Fig. 5a). However, the observed distribution of unique vs. shared replicon sequences between the two continents was not statistically significant (Fisher’s exact test, *p* = 1).

A total of 28 MAGs from European (n = 11) and African (n = 17) stools were associated with plasmids co-carrying replicon sequences and ARGs (Fig. 5b). In particular, European stools frequently harbored rep1 and repUS43 replicon types of Gram-positives carrying ARGs such as *aac(6’)-aph(2”)*, *erm(B)*, and *tet(M)*, possibly hosted by *Enterococcus* species (Fig. 5b; Table S4). They were also associated with the IncF family of Gram-negatives carrying, for example, *bla*_CTX−M−15_ and *bla*_TEM−1D_, typically present in *Enterobacteriaceae* (e.g., *E. coli*). In contrast, African stools often contained the repUS43 from *Enterococcus faecium* plasmids carrying only *tet(M)*, as well as distinct IncF-type plasmids possessing either single ARGs (e.g., *bla*_CTX−M−15_) or multiple ARGs [e.g., *aph(6)-Id*, *aph(3”)-Ib*, *aadA5*] predicted to be hosted by archetypal *Enterobacteriaceae* (e.g., *E. coli*,* Klebsiella pneumoniae*) (Fig. 5b; Table S4).

## Discussion

There is growing interest in understanding how microbiota dynamics shift in response to novel environments, such as exposure to regions of high AMR burden (e.g., African continent)^25^. These changes are of epidemiological importance, as they may reveal links between microbiota alterations and potential vehicles of ARG dissemination [e.g., plasmids^27^ that are often overlooked by traditional single-strain intestinal colonization studies, which typically focus on specific bacterial types (e.g., *Enterobacteriaceae*)]^16^. In contrast, untargeted SMS approaches may provide a broader and more accurate representation of the microbiota and the resistome composition, allowing for comprehensive intestinal colonization studies.

### Minimal microbiota alterations in Swiss expats at return from abroad

We recently demonstrated that Swiss expatriates living in African countries often return to Switzerland colonized at the intestinal level with MDR-Ent^15^. Similarly, several studies have shown that travel to African countries is associated with a risk of becoming colonized with MDR-Ent. However, few of them have addressed how travel to the African continent may impact the overall composition of the gut microbiota, an important aspect for advancing our understanding of the microbiome (e.g.,^1,2,4,5^).

For instance, a recent metagenomic study by Cheung et al. analyzed stool samples from 90 healthy Chinese travelers and reported no significant changes in microbiota composition (both alpha- and beta-diversity) before and after travel^1^. Likewise, Bengtsson-Palme et al. (2015) studied 35 Swedish students before and after exchange programs, including those in Central Africa, and found no differences in taxonomic composition, apart from an increased abundance of Proteobacteria in some students^2^. In contrast, Worby et al.^5^ observed altered microbiota composition (Shannon diversity) and increased *Enterobacteriaceae* abundance in travelers from the USA to African countries (89 out of 267 participants), particularly those visiting Southern and West Africa^5^. Notably, all these studies employed Illumina-based shotgun metagenomic sequencing, with sequencing depths ranging from 14 million (M) to 463 M paired-end reads.

In contrast to the above-mentioned studies, despite key differences in study design (e.g., longitudinal vs. cross-sectional), sequencing depth [mean of 409 thousand (T) long reads] and platforms, our Nanopore-based metagenomic study revealed some notable similarities. Specifically, when comparing Swiss expatriates living in African countries to those residing in other European countries, we also observed no significant differences in microbiota composition, as shown by abundance and diversity analyses (Figs. 1 and 2, and S3).

These data suggests that intestinal colonization with bacteria such as MDR-Ent may occur independently without influencing dramatic microbiota composition changes. We also note that the presence of a higher number of colonization-positive samples among individuals in African countries compared to those in Europe (19/39 vs. 4/33, respectively), did not result in alteration to the overall microbiota composition (Table S1; Figs. 2 and S3). This indicates that factors such as foreign country of residence, duration of stay, and MDR-Ent colonization status did not appear to drive substantial differences in the gut microbiota of returning expats. These findings are in line with a Dutch study of healthy adults, which found that intestinal colonization, particularly with ESBL-producing *E. coli*, does not result in significant changes to the microbiota^28^, although another study in Thailand reported the opposite^29^. We previously also showed that the microbiota of travelers to India does not significantly change over time regardless of colonization status^30^. Overall, these studies suggest a more complex scenario likely influenced by differences, for example, in the populations studied or sequencing approaches.

### Resistome differences at return from European versus African countries

Equally important, but perhaps more relevant in an epidemiological AMR context, is the characterization of the resistome following exposure to high-endemic regions. Few studies have focused their efforts on characterizing not only the microbiota, but also the resistome of healthy people, particularly after traveling to African countries (e.g.,^1–5^).

In the present study, we identified minor but important differences in the relative abundance of ARGs between African and European stools. In particular, we found that African stools contained a larger proportion of tetracycline-encoding ARGs (*tet*), as well as those predicted to encode for folate pathway antagonists (*dfr*, *sul*), whereas ARGs for macrolides (*erm*) were more prevalent in European stools (Figs. 3 and 4; Table 1). Likewise, we found that *tet* and *erm* ARGs were likely carried by *Ruminococcus* (or *Ruminococcoides* as per GTDB-Tk), *Bifidobacterium*, and *Bacteroides* genera in European and African stools (Table S3; Fig. 1; Supplementary Data 2). We note that *tet* genes are prevalent in the human gut, especially in *Ruminococcus* and *Bifidobacterium*, while *erm* genes are more commonly found in *Bacteroides*, consistent with the natural resistome^31^.

We also identified important β-lactam encoding genes such as oxacillinases and AmpCs (e.g., *bla*_OXA−1_ and *bla*_ACT−6_, respectively) in African stools, including the clinically relevant ESBL *bla*_CTX−M−15_ in both European and African stools (Fig. 5; Table 1; Supplementary Data 1–3). In particular, *bla*_TEMs_ and *bla*_CTX−M−15_ were associated to *E. coli* genomes, consistent with our culture-based findings (Table S1; Table S3; Supplementary Data 2)^15,17,18^.

Consistent with these results, other studies have also reported an increased number of ARGs, including *tet* genes and high-risk AMR gene families (e.g., *dfr*, *qnr*, *bla*_CTX−M_), following travel to countries on the African continent^1–3,5^. We note that in these studies, the reported travel duration varied, ranging from a median of 13 to 35 days. As a result, these observations cannot be directly compared to our study, which involved Swiss expatriates residing abroad for ≥ 3 months, and in their current city for a median of 1.4 years (interquartile range: 1.0-2.6) (Table S1)^15^. Moreover, the stability of the resistome following international travel (or residing abroad) remains a controversial subject, raising questions about whether the observed changes are truly representative or simply a temporal effect^5,32^.

It is also important to understand the key drivers potentially influencing such resistome changes. These may include regional differences in antibiotic use or consumption patterns (e.g., higher usage of trimethoprim-sulfamethoxazole and tetracyclines in parts of Africa, and more frequent macrolide use in Europe)^25,33,34^. These patterns are reflected in the predominant ARGs identified in this study (e.g., *dfr* and *tet* genes in Africa; *erm* genes in Europe) (Figs. 3 and 4; Table 1). Taken together, our results underscore that resistome variation among returning expatriates cannot be generalized solely on the basis of the foreign country of residence, but rather interpreted in the context of length of stay and endemic microbial and antimicrobial exposure.

### The plasmidome as a source of antimicrobial resistance determinants

Plasmids are among the most critical MGEs responsible for the global dissemination of dangerous ARGs^27^. Their association with epidemiologically and clinically relevant ARGs is of particular interest, as plasmids can, for instance, encode carbapenemases (e.g., KPC, NDM), ESBLs (e.g., CTX-Ms), and quinolone resistance determinants (e.g., Qnr), ultimately limiting our therapeutic armamentarium^27,35,36^. While their impact and spread are typically studied in intestinal colonization prevalence surveys (e.g.,^6–14,37^), they are often overlooked in metagenomic studies due to the need for long-read sequencing data to accurately determine the *de novo* location of ARGs and their surrounding genomic elements.

With the implementation of Nanopore-SMS, we were able to partially resolve putative plasmids (i.e., contigs containing only replicon sequences) and those associated with ARGs directly from metagenomic data. For instance, the repUS43 plasmid carrying only the *tet(M)* gene was more frequently observed in African than European stools (11/13 vs. 2/13, respectively) (Fig. 5; Table S4). Interestingly, this plasmid is commonly associated with *Enterococcus* spp. and has been linked to the chicken metagenome^38^. It has also been recovered from diverse environments, including wastewater in Brazil, patient samples and chicken litter in South Africa, suggesting a strong link within the One Health context^39,40^.

We also identified well-known and epidemiologically relevant plasmid replicon types (i.e., those capable of conjugation in Ent) in both European and African stools. Many of them carried multiple ARGs previously reported in *E. coli* or other *Enterobacteriaceae* from human, animal and food sources, such as the IncB/O/K/Z-, IncF-, IncQ-, and hybrid-type plasmids (both IncF- and IncQ-type)^41,42^ (Fig. 5; Table S4). Two IncF-type plasmids were associated with *bla*_CTX−M−15_ or co-localized with *qnrS1*, a finding consistent with previous data from single-strain WGS (Illumina and Nanopore) and targeted Nanopore-SMS pre-enrichment (see below)^17,18^. Notably, the *bla*_CTX−M−15_ and *qnrS1* IncFII plasmid was recently reported in azithromycin-resistant *Shigella flexneri* 1b from shigellosis cases in Ontario, Canada^43^, while the IncFIB(H89-PhagePlasmid) carrying only *bla*_CTX−M−15_ resembles a bacteriophage-like plasmid first described from a human clinical specimen^44^. We note that this plasmid has also been recovered from the hyperepidemic *E. coli* sequence type (ST) 131 in wastewater and environmental water in Switzerland^45^.

Importantly, none of the metagenomic studies discussed above (e.g.,^1–5^) linked ARGs to plasmids. Only the study by Cheung et al., involving healthy Chinese international travelers, reported ARGs such as *tet* and *qnr* families associated with *Escherichia*/*Shigella* bins^1^. Linking ARGs to taxa may be achieved through metagenomic binning, which involves grouping MAG contigs into taxonomic bins to infer links between microbial identity and ARGs^19^. In our study, we associated most non-plasmid-linked ARGs with genera of the class Clostridia (e.g., *Blautia*, *Faecalibacterium*), which were also among the most relative abundant genera (Fig. 1; Table S3).

These findings highlight the limitations of short-read metagenomic studies, whereas long-read approaches like ours can go further by resolving MGEs - specifically ARG-carrying plasmids - as demonstrated in recent clinical metagenomic studies (e.g.,^17,20–23^), including those focused on African microbiota and resistome populations (e.g.,^23,26^), thus providing both taxonomically linked and mobile resistome profiles with improved resolution.

### Suitability of nanopore-SMS to study the microbiota and resistome

It is important to note that while Nanopore-SMS offers advantages such as real-time sequencing and long-read capability, it typically yields lower sequencing depth than Illumina-based methods. In this study, our amplification-free Nanopore metagenomic approach generated a mean of ~ 409 T reads, significantly lower than Illumina-based studies (e.g., 14–463 M paired-end reads)^1,2,4,5^. Nonetheless, we believe this depth was sufficient to describe the microbiota and resistome, though it potentially limited deep microbiota profiling, aligning with findings from studies on international travelers to the African continent^1,2,4,5^. Still, this reduced depth must be considered when interpreting the microbiota and resistome composition, as it may limit the detection of low-abundance ARGs or rare taxa.

Future comparative studies should also explore alternative Nanopore-SMS strategies, such as the rapid metagenomic sequencing protocol using the SQK-RPB114.24 kit by Nanopore (nanoporetech.com/document/rapid-sequencing-metagenomics-sqk-rpb114-24). Alternatively, combining short- and long-read technologies, as recently applied in population-level African studies^23,26^, could greatly enhance sequencing depth and thus produce a more robust microbiota profiling. Notably, hybrid SMS (Illumina and Nanopore) approaches may also improve the depth of the resistome, assisting the resolution of plasmids, including those carrying ARGs^24^.

Importantly, metagenomic studies aimed at detecting clinically and epidemiologically relevant ARGs (e.g., those encoding ESBLs and carbapenemases) must account that some bacteria (e.g., MDR-Ent) and their associated ARGs may be present in native stool at concentrations below the detection limit^18^. To overcome this limitation, targeted pre-enrichment approaches are necessary to enhance the resolution of important resistance-associated bacteria and their ARGs, as we have previously demonstrated using both Illumina and Nanopore platforms^17,18^. On the other hand, because targeted pre-enrichment may introduce bias, particularly in the context of microbiota and resistome profiles due to antibiotic selection pressure — native stool screening, as performed in this study and others above^1–5^, remains the most appropriate method for unbiased profiling of both microbiota and resistome. Finally, since SMS provides a snapshot of all the present DNA of all organisms (e.g., bacteria) in a sample, whether dead or alive, metagenomic surveys should include validation of key findings using culture-based methods (e.g.,^2–5,17,18^), particularly when clinically relevant ARGs (e.g., *bla*_CTX−Ms_) are detected, to strengthen the epidemiological validity of such studies.

### Conclusions

 Nanopore-SMS enables comprehensive characterization of the microbiota — in particular the resistome — and may be well-suited for preliminary survey investigations of people living abroad in endemic regions who are at risk of intestinal colonization by bacterial pathogens and their associated ARGs. Importantly, our findings underscore that resistome studies should consider the plasmidome as a key reservoir contributing to the potential spread of AMR. The results of such metagenomic studies can serve as a foundation for targeted epidemiological investigations. These findings highlight the need for further research into at-risk and under-represented populations (e.g., expatriates), as well as the long-term health implications of microbiome and resistome changes within a broader One Health context.

## Methods

### Study design and stool sample selection

A total of 72 previously characterized stool samples were analyzed. They were collected in Switzerland upon the return of healthy volunteers (one stool sample per volunteer) who had been residing in countries within the European (*n* = 33) and African (*n* = 39) continents^15,17,18^. All 72 participants, who were ≥ 18-year-old and living abroad (≥ 3 months) provided informed written consent, were part of an on-going prospective prevalence study on intestinal colonization of Swiss expats (data.snf.ch/grants/grant/192514).

Stool samples were self-collected in preservative-free containers (Thermo Fisher Scientific), stored at 4 °C, mailed within 7 days of return to Switzerland for processing in our institute (IFIK, University of Bern, Switzerland) as previously described^15^. In brief, stool specimens were screened for extended-spectrum cephalosporin-resistant Enterobacterales by broth pre-enrichment for 6 h at 36 ± 1 °C in Luria-Bertani broth containing cefuroxime (3 mg/L), followed by overnight plating on selective ChromID ESBL (bioMérieux) agar plates^15,18^. Bacterial colonies grown on the overnight plates were selected for species identification using the matrix-assisted laser desorption/ionization time-of-flight mass spectrometry (MALDI-TOF MS). Antimicrobial susceptibility tests were performed by the broth microdilution method using the Sensititre GNX2F panels (Thermo Fisher Scientific). Strains were subjected to WGS, as previously described^15,17,18^. A summary of the previously described characteristics of the 72 stools selected for this study — including representative demographics, risk factors, culture-based intestinal colonization screening results, strain phenotype and genotype characteristics — is shown in Table S1^15,17,18^.

### Stool genomic DNA (gDNA) isolation and metagenomic sequencing

In brief, ~ 200 mg aliquots per stool sample stored at −80 °C were subjected to gDNA extraction using the QIAamp PowerFecal Pro DNA Kit (QIAGEN) following manufacturer recommendations. The quality and concentration of the resulting gDNA isolations were assessed by NanoDrop and Qubit 3 (Thermo Fisher Scientific).

The Nanopore SQK-RBK114-24 kit using 200 ng of input gDNA was used to prepare sequencing libraries following manufacturer recommendations (protocol version: RBK_9176_v114_revP_27Dec2024). Each sample (i.e., stool gDNA) was run in duplicate and randomly assigned to 7 separate sequencing libraries, which were loaded onto R10.4.1 flow cells on a MinION Mk1B device. Sequencing run data acquisition and demultiplexing was done on the MinKNOW software (v24.11.8) for 72 h with the options minimum read quality score (Q-Score) set to 10, barcode splitting to “ON”, and basecalling to super-accurate (r1041_e82_400bps_sup_v4.3.0) mode with Dorado v7.6.7. All basecalled reads (i.e. multiple FASTQ files) belonging to the same barcode were concatenated into a single file before downstream processing.

### Nanopore-read preprocessing

Nanopore sequencing adaptor and barcode sequences were trimmed from FASTQ files with Porechop v0.2.4 (github.com/rrwick/Porechop) using the parameter ‘--discard_middle’. Thereafter, FASTQ files were decontaminated from host reads using the human reference genome GRCh38.p14 (RefSeq accession: GCF_000001405.40) as previously described^17^. At each preprocessing step (post sequencing, trimming, and host decontamination), sequencing read statistics were determined with NanoStat v1.4.0 (github.com/wdecoster/nanostat) with default parameters. Lastly, before downstream analyses, all host-decontaminated FASTQ files from the same stool sample (i.e., randomly sequenced in duplicates) were concatenated with the linux command-line tool “cat” into a single FASTQ file per sample to maximize sequencing depth.

### Read-based taxonomic classification, abundance and diversity analyses

Taxonomic classification of host-decontaminated reads was done at the genus level with Kraken2 v2.1.4 (pre-built index: K2 standard (12/28/2024); https://benlangmead.github.io/aws-indexes/k2) with the following arguments: ‘--use-names --report-zero-counts --confidence 0.05’^46^. Thereafter, the Kraken2 reports of bacterial taxonomy (all ranks) were merged and processed in the R programming language v4.4.1 with the phyloseq v1.48.0 package as previously described^17^. In phyloseq, the merged Kraken2 report was preprocessed at the genus level. Taxa with zero or single counts across all samples, as well as those present in fewer than two samples, were removed. The preprocessed taxonomic read count table was normalized by subsampling without replacement in phyloseq (‘rarefy_even_depth’; rngseed= ‘1718’, replace= ‘FALSE’) to account for unequal sequencing depth before relative abundance and diversity analyses (see below)^47,48^. The rarecurves were generated with ‘rarecurve’ from the vegan v2.6-8 R package.

Relative abundance analysis was done in phyloseq with the ‘transform_sample_counts’ function using the subsampled dataset stratified by continent and showing the top 15 genera. Moreover, the Shannon, Simpson and Observed diversity indices were estimated on the subsampled dataset in phyloseq with the ‘estimate_richness’ function. Beta diversity analyses were done in phyloseq using the subsampled dataset transformed to proportions (function; ‘transform_sample_counts’). In phyloseq, the Bray-Curtis distances were calculated using the ‘distance’ function, followed by ordination based on non-metric multidimensional scaling (NMDS) using the ‘ordinate’ function. Beta dispersion of the data stratified by continent was then calculated using the ‘betadisper’ function from the vegan R package.

For all the above analyses and those described below, data wrangling and plotting were performed in R using the tidyverse v2.0.0 and ggpubr v0.6.0 packages, with stylistic enhancements made in Adobe Illustrator CS6 v16.0.3 (64-bit).

### Generation of meta-assembled genomes (MAGs)

Generation of MAGs were done as previously described^17^. In brief, the human-decontaminated reads (described above) were assembled with metaFlye v2.9.5-b1801 with the arguments ‘--nano-raw --meta’. The raw assemblies were polished 4 rounds with Racon v1.5.0 (default parameters) (https://github.com/lbcb-sci/racon), followed by one last polish round with Medaka v2.0.1 (https://github.com/nanoporetech/medaka) using the ‘medaka_consensus’ command and model (-m) ‘r1041_e82_400bps_sup_v4.3.0’ argument.

### ARG screening and abundance Estimation

The polished MAGs were screened for ARGs with ResFinder v4.6.0 bioconda package (https://anaconda.org/bioconda/resfinder) using the arguments ‘-ifa --acquired’ and default identity threshold (90%) and minimum length (60%). Read counts were determined with a custom Python v3.9.19 script. In brief, the output file ‘ResFinder_Resistance_gene_seq.fsa’ containing the ARG sequence hits from the ResFinder screen was used to map nanopore reads with minimap2 v2.28-r1209 (arguments: ‘-ax’ ‘map-ont’). Read counts were then extracted with samtools v1.21 using the command ‘view’ and the arguments ‘-F 4’.

The ARG read counts were imported and analyzed in phyloseq for easy handling and plotting. Prior to downstream analyses, ARG read counts were normalized using the TPM method. Briefly, raw counts were divided by ARG gene lengths in kilobases to obtain reads per kilobase (RPK), then scaled by the sum of RPKs per sample and multiplied by a million to account for sequencing depth, enabling appropriate between-sample comparisons^49^. The predicted ARG classes were manually curated from the ResFinder database Bitbucket repository (https://bitbucket.org/genomicepidemiology/resfinder_db/src/master/) ‘phenotypes.txt’ file (see Supplementary Data 1 for the defined ARG classes used in this study).

Relative abundance analysis of ARGs was done in phyloseq with the ‘transform_sample_counts’ function using the TPM normalized dataset stratified by continent and showing the proportions of ARG phenotypes. Log 10 transformed TPM counts were used to generate standard boxplots of each predicted ARG phenotype. The Bray-Curtis-based principal coordinates analysis (PCoA) ordination of TPM-normalized ARG counts was conducted in phyloseq as described above. Lastly, differences in unique and shared ARGs between groups (Europe vs. Africa) were examined to assess whether the number of unique ARGs per class varied between continents.

### Plasmid replicon-typing and linking to ARGs

The polished MAGs were screened for the presence of plasmid replicon sequences using PlasmidFinder v2.1.6 bioconda package (https://anaconda.org/bioconda/plasmidfinder) with default identity threshold (95%) and minimum length (60%). Plasmid replicon sequence counts were obtained using a custom Python script, based on the ‘Plasmid_seqs.fsa’ output file, which contained replicon sequence hits identified by the PlasmidFinder screen (as described in the ARG screening section above).

A plasmid was defined when a MAG contig contained at least one replicon sequence, consistent with PlasmidFinder *in silico* replicon typing^50^. ARGs were considered linked to plasmids when both ARGs and replicon sequences were co-located on the same MAG contig. In such cases, co-location of ARGs and replicon sequences was determined by matching the ResFinder and PlasmidFinder outputs files with a custom Python script, respectively. Furthermore, MAG contigs co-carrying ARGs and replicon sequences were manually screened with the PLSDB plasmid database (version 2024_05_21_v2) online platform (https://ccb-microbe.cs.uni-saarland.de/plsdb2025/) using mash screen with the options of max *p*-value set to 0.1 (default), minimum identify to 0.95, and ‘Winner-takes-all strategy’ option. In addition, results were supplemented with a custom nucleotide BLAST v2.16.0 + with the PLSDB plasmid database described above.

The plasmid replicon sequence read counts were imported and analyzed in phyloseq for easy handling and plotting. The Venn diagram of unique and shared replicon sequences between Europe and Africa was done with ggvenn v0.1.10 R package. An alluvial diagram was generated using the ggalluvial v0.12.5 R package to illustrate the flow between continent, plasmid class, plasmid type, and ARGs in samples where MAGs carried co-located ARGs and plasmid replicon sequences within the same contig (see definition above).

### Taxonomic determination of MAGs with ARGs not linked to plasmids

The polished MAGs were binned with MetaBat2 v2.17 (https://bitbucket.org/berkeleylab/metabat/src/master/). The resulting MAG bins were improved and dereplicated with DAS Tool v1.1.7 (https://github.com/cmks/DAS_Tool) with the argument ‘--score_threshhold’ set to 0.5 (default). Then, the quality and the taxonomic composition of the dereplicated bins was estimated with CheckM v1.1.2 (https://github.com/Ecogenomics/CheckM) and contextualized with the read-based taxonomic classification results with Kraken2 described above. Lastly, high-quality MAG bins as determined by CheckM (> 90% completeness and < 5% contamination) were taxonomically classified with the genome taxonomy database tool kit (GTDB-Tk) v2.4.1 (https://github.com/Ecogenomics/GTDBTk) using GTDB release R226 (command: ‘classify_wf’ and ‘--mash_db’).

### Statistical analyses

Statistical analyses were conducted in the R programming language. Datasets with a normal distribution and equal variance were compared using an unpaired, two-sided t-test. For non-normally distributed data, the Wilcoxon Rank Sum test (unpaired; two-sided) was applied. Where appropriate, an exact permutation version of the Wilcoxon Rank Sum test was used (via the coin R package: exact Wilcoxon-Mann-Whitney test), which calculates *p*-values based on the exact distribution generated by all possible permutations of group labels. This approach was implemented in cases where tied or zero-inflated data violated assumptions of the standard asymptotic method. The ‘permutest’ function (permutations = 999) from the vegan R package was used to test for homogeneity of multivariate dispersions. The PERMANOVA test (using adonis2 from the vegan R package) was used to assess whether differences in microbiota and resistome composition, based on Bray-Curtis distances, could be explained by continent. The Fisher’s exact test (two-sided) performed with the stats v4.4.1 R package was used to assess the significance in distribution between unique vs. shared ARGs and plasmid replicon sequences between Europe and Africa. Results from the multiple Fisher’s exact ARG tests were adjusted for multiple comparisons using the false discovery rate (FDR) correction.

## Supplementary Information

Below is the link to the electronic supplementary material.

Supplementary Material 1

Supplementary Material 2

Supplementary Material 3

Supplementary Material 4

## Data Availability

The human decontaminated and preprocessed Nanopore reads (two runs per sample; *n*=144) were deposited in the sequence read archive (SRA) under BioProject accession PRJNA1285875. The MAGs generated in this study, along with intermediary analysis outputs (Kraken2, ResFinder, PlasmidFinder, CheckM, and GTDB-Tk reports), are available in Zenodo ( https://doi.org/10.5281/zenodo.15845671 ).
