# Supplementary material for: Gut microbiota and resistome profiles of Swiss expatriates in Africa revealed by Nanopore metagenomics
Source: Sci Rep. 2026 Feb 3;16:7016. doi: 10.1038/s41598-026-38302-3 (PMC12920924; doi:10.1038/s41598-026-38302-3)
Supplement: Supplementary file 1 — Supplementary Material 1 [file 41598_2026_38302_MOESM1_ESM.pdf]

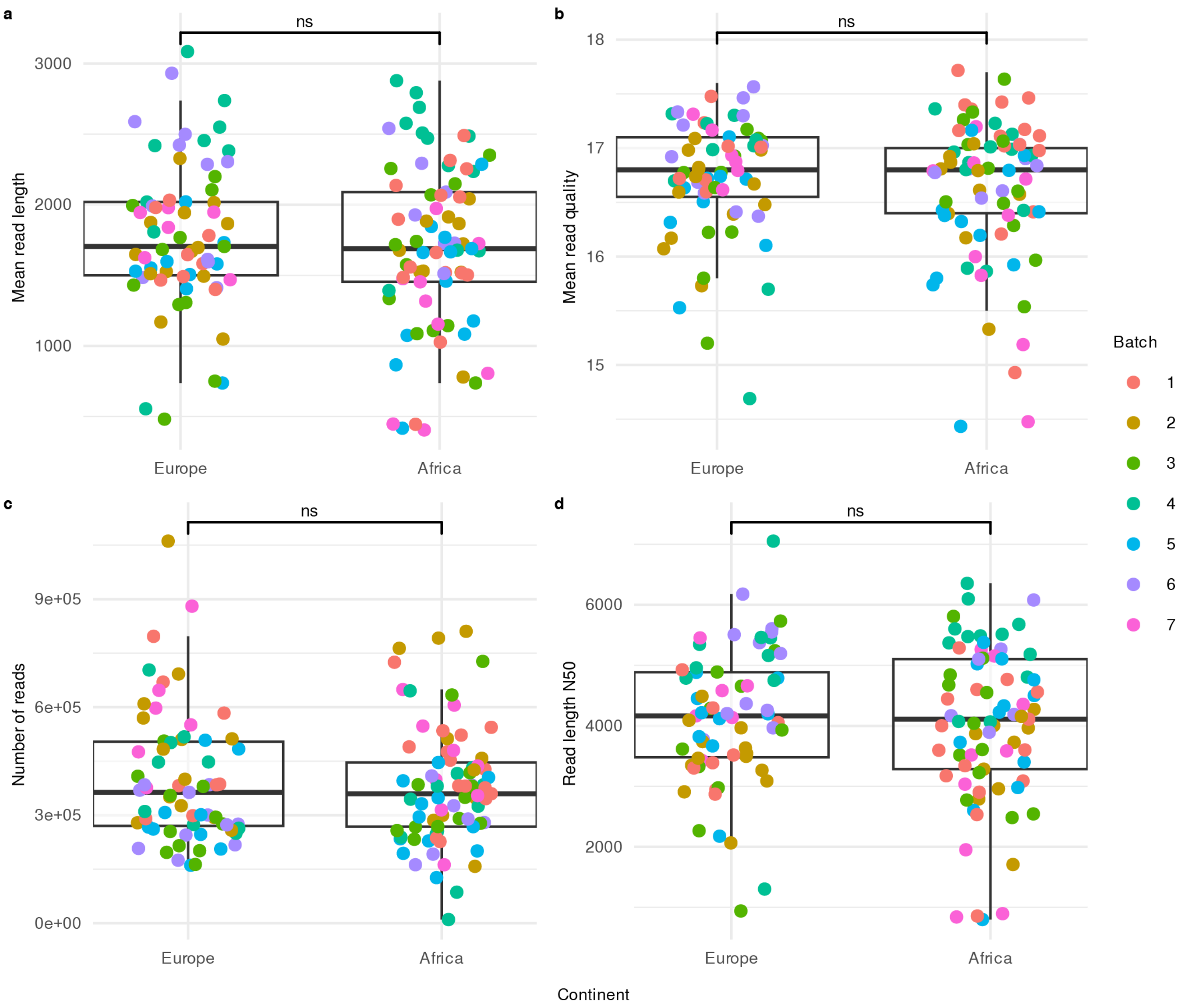

Figure S1. Sequencing output metrics of Nanopore-SMS after preprocessing (barcode trimming and host decontamination) of European (n=33) and African (n=39) stool samples run in duplicate (n=66 and 78, respectively) in 7 random sequencing batches. The sample distribution for each sequencing metric is shown in the boxplots stratified by continent: **a**) mean read length (bp, base pairs), **b**) mean read quality (Q-Score), **c**) number of reads, and **d**) read length N50 (bp). In all boxplots, the line in the center of the box represents the median, while the 25th and 75th percentiles are the lower and upper bounds of the box, respectively. Lower and upper whiskers extend the box, representing data points outside the interquartile range (1.5 times). Statistical significance between groups (Europe *vs*. Africa) is indicated by the significance level marked above the bracket (ns, not significant). The unpaired non-parametric Wilcoxon rank sum test (two-sided) was used to compare groups in boxplot **b-d**, while an unpaired Student's t-test (two-sided) was used to compare groups in boxplot **a**. The exact *p*-values for boxplots **a**, **b**, **c**, and **d** are 0.36, 0.49, 0.77, and 0.72, respectively. Source data are provided as a Source Data file.

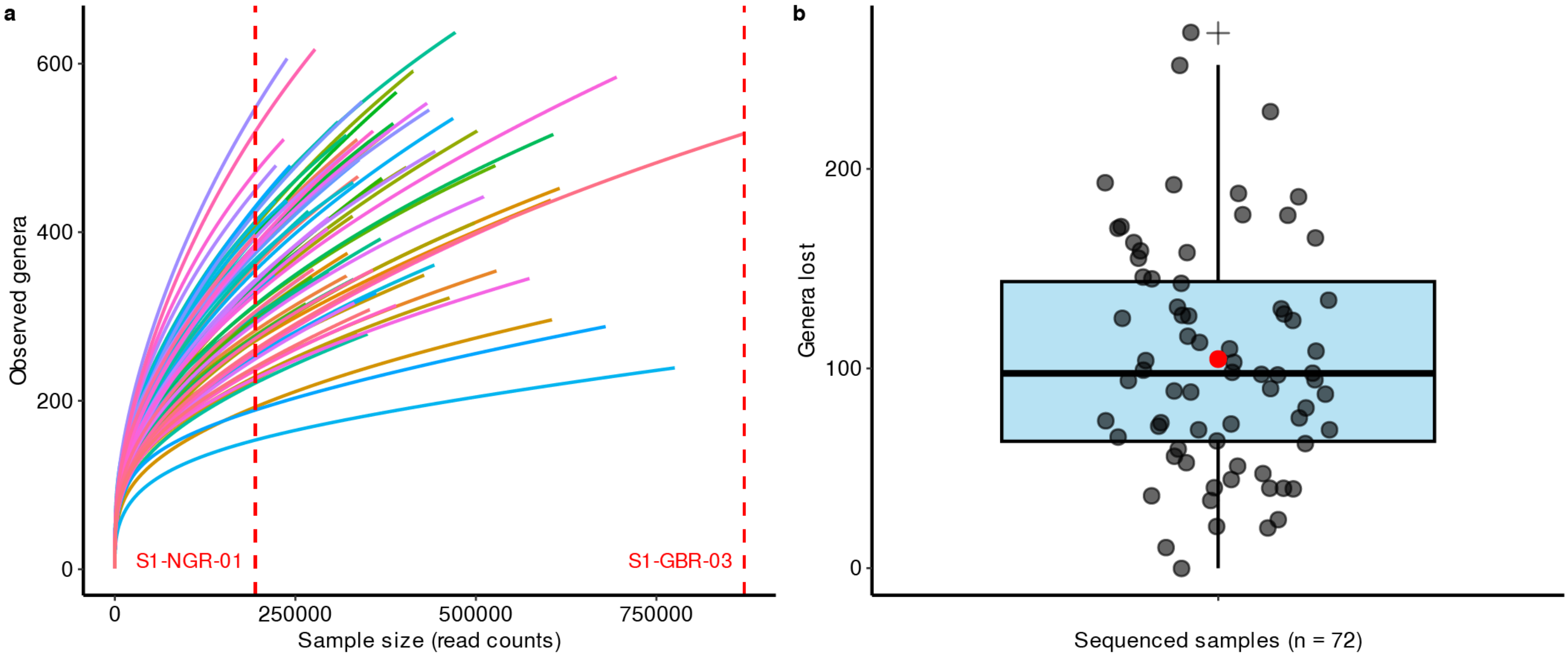

Figure S2. Normalized read counts by subsampling before downstream taxonomic analyses at the genus level. Figure **a)** shows the number of observed genera counts subsampled at various sampling depths across all samples (represented by colored lines). The samples with the smallest and largest total read counts are shown in red with vertical dashed lines. In **b)** we show the distribution of genera lost across all samples after subsampling. In the boxplot, the red circle and line in the center of the box represent the mean and the median, respectively. The 25th and 75th percentiles represent the lower and upper bounds of the box, respectively. Lower and upper whiskers extend from the box, representing data points outside the interquartile range (1.5 times). The cross symbol extending the upper whisker represents an extreme outlier. Source data are provided as a Source Data file.

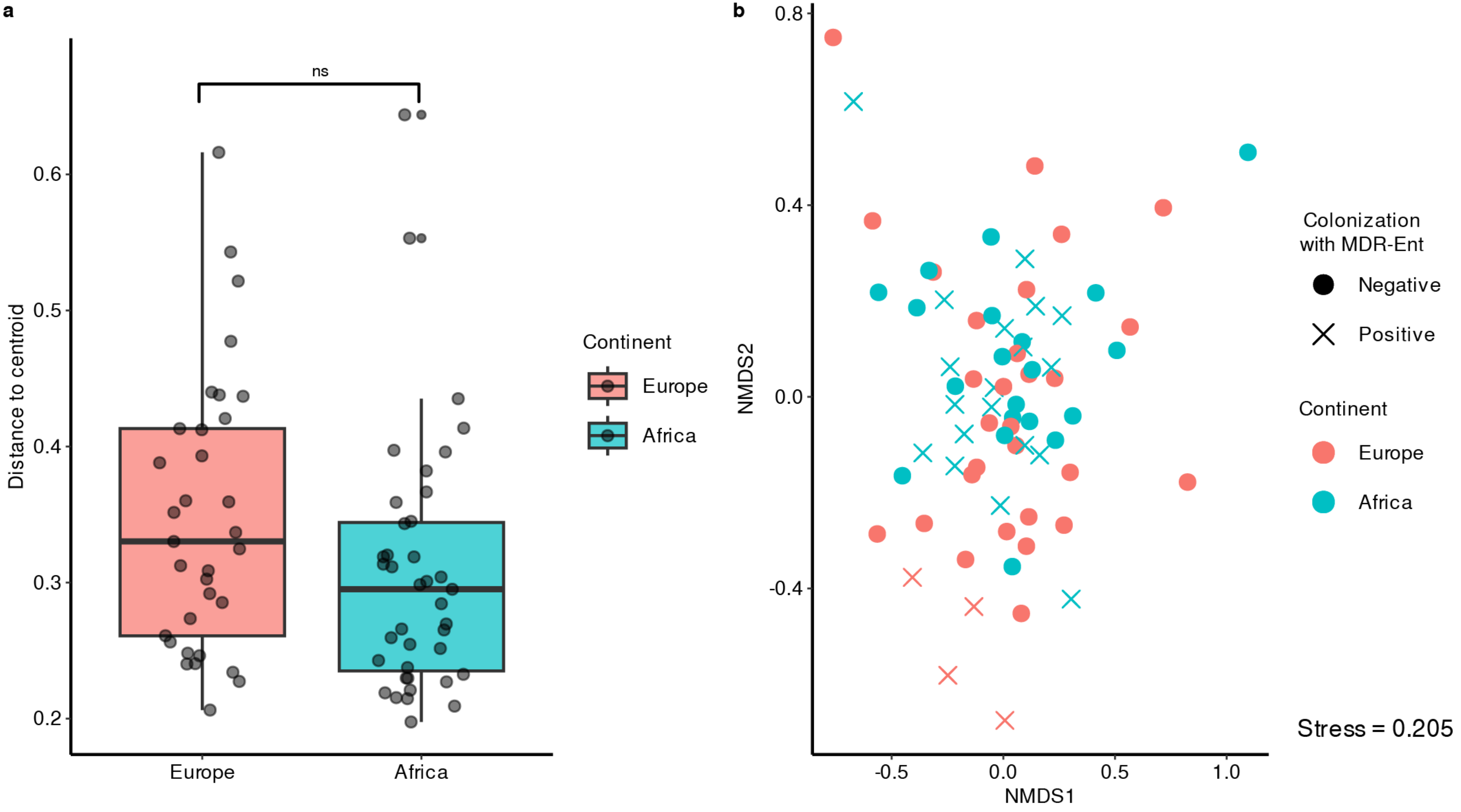

Figure S3. Beta dispersion and dimensionality reduction analyses performed on the Bray-Curtis distance of European (n=33) and African (n=39) stool samples using a subsampled, proportion-transformed dataset. The boxplots (**a)** display the distance to centroid for all samples, stratified by continent. In the boxplots, the line in the center of the box represents the median, while the 25th and 75th percentiles are the lower and upper bounds of the box, respectively. Lower and upper whiskers extend the box, representing data points outside the interquartile range (1.5 times). Statistical significance between groups (Europe *vs*. Africa) is indicated by the significance level marked above the bracket (ns, not significant). The permutest (permutation test for homogeneity of multivariate dispersions) was used to compare groups in boxplots **a.** The exact *p*-value for plot **a** is 0.073. In plot **b**) we show a non-metric multidimensional scaling (NMDS) ordination using the Bray-Curtis distance. Circles are colored by continent, while intestinal colonization with MDR-Ent status is represented by a circle or an ‘X’ for negative or positive, respectively. The stress value is provided in the plot. Source data are provided as a Source Data file.

Table S1. Summary of representative demographics, risk factors, culture-based results, strain phenotype and genotype characteristics of the study samples.

Stool sample	Representative demographics and risk factors [a]												Culture-based intestinal colonization screening [b]		Strain genotype [c]	
	Continent	Country	Gender	Age	Time (years) living abroad in current country	Lived in other countries in the last 5 years	Hospitalizations during the last 2 years	Antibiotic used during the last year	Chronic disease(s) affecting the gastro-intestinal tract	Constantly taking stomach antacids or acid reducers	Experienced diarrhea in the last 3 months	Diet	Result	Phenotype of the isolated *E. coli* strain	Resistance mechanism	*E. coli* ST
S1-AUT-01	Europe	Austria	Female	46	1	Yes	No	No	Yes	No	No	Omnivore	Negative	-	-	-
S1-BEL-01	Europe	Belgium	Female	53	31.8	No	No	No	No	No	No	Omnivore	Negative	-	-	-
S1-CAM-05	Europe	Switzerland	Male	57	0	Yes	Yes	Yes	No	No	No	Omnivore	Negative	-	-	-
S1-CHI-01	Europe	Switzerland	Female	56	1.9	Yes	No	Yes	No	No	No	Omnivore	Positive	3GC-R	CTX-M-15	ST69
S1-COD-01	Europe	Switzerland	Male	46	15.6	Yes	No	No	Yes	No	Yes	Omnivore	Negative	-	-	-
S1-DNK-01	Europe	Denmark	Female	58	2.7	Yes	No	No	No	No	No	Omnivore	Negative	-	-	-
S1-ESP-01	Europe	Spain	Female	39	12.3	No	No	Yes	Yes	No	No	Omnivore	Negative	-	-	-
S1-ESP-02	Europe	Spain	Female	25	1.4	No	No	Yes	No	No	Yes	Vegan	Positive	3GC-R	CTX-M-15	ST1193
S1-ESP-03	Europe	Spain	Female	51	4.2	Yes	No	No	No	No	Yes	Omnivore	Positive	3GC-R	CTX-M-15	ST69
S1-GBR-01	Europe	Great Britain	Female	55	1.3	Yes	No	Yes	No	No	Yes	Omnivore	Negative	-	-	-
S1-GBR-03	Europe	Great Britain	Male	57	1.5	Yes	No	No	No	No	No	Omnivore	Negative	-	-	-
S1-GER-01	Europe	Germany	Male	54	3.3	No	No	No	Yes	No	No	Vegetarian	Negative	-	-	-
S1-GER-02	Europe	Germany	Male	56	3.3	Yes	No	No	No	No	No	Vegetarian	Negative	-	-	-
S1-GER-03	Europe	Germany	Male	34	4	Yes	No	No	No	No	No	Omnivore	Negative	-	-	-
S1-GER-04	Europe	Germany	Female	32	2	Yes	No	No	No	No	No	Omnivore	Negative	-	-	-
S1-GER-05	Europe	Germany	Male	63	3.1	No	No	No	No	No	No	Omnivore	Negative	-	-	-
S1-GER-06	Europe	Germany	Female	60	3.1	No	No	No	No	No	No	Vegetarian	Negative	-	-	-
S1-GER-08	Europe	Germany	Female	27	0.3	No	No	No	No	No	No	Omnivore	Negative	-	-	-
S1-IND-06	Europe	Switzerland	Female	41	0.5	Yes	No	No	No	No	Yes	Vegetarian	Negative	-	-	-
S1-ITA-01	Europe	Italy	Female	31	0.4	Yes	Yes	No	No	No	Yes	Omnivore	Negative	-	-	-
S1-KOR-01	Europe	Switzerland	Female	35	8	No	Yes	No	No	No	Yes	Omnivore	Negative	-	-	-
S1-KOS-01	Europe	Kosovo	Male	47	11.5	Yes	No	No	No	No	No	Omnivore	Negative	-	-	-
S1-KOS-02	Europe	Kosovo	Female	38	0.4	Yes	No	Yes	No	No	No	Omnivore	Negative	-	-	-
S1-KOS-03	Europe	Kosovo	Male	60	3.2	Yes	No	No	No	No	Yes	Omnivore	Negative	-	-	-
S1-NED-01	Europe	Netherlands	Female	31	4.6	No	No	No	No	No	No	Vegetarian	Negative	-	-	-
S1-POR-01	Europe	Portugal	Female	45	1.5	Yes	Yes	No	No	No	No	Omnivore	Negative	-	-	-
S1-SRB-01	Europe	Serbia	Male	62	1.3	Yes	No	No	No	Yes	No	Omnivore	Negative	-	-	-
S1-SRB-02	Europe	Serbia	Female	62	1.2	Yes	No	Yes	No	No	No	Omnivore	Positive	3GC-R	CTX-M-15	ST69
S1-THA-03	Europe	Switzerland	Female	60	2.4	Yes	No	No	No	No	No	Omnivore	Negative	-	-	-
S1-UKR-01	Europe	Ukraine	Female	35	0.6	Yes	No	Yes	Yes	No	No	Omnivore	Negative	-	-	-
S1-VEN-02	Europe	Switzerland	Male	43	1.3	Yes	No	No	No	No	No	Omnivore	Negative	-	-	-
S1-VEN-03	Europe	Switzerland	Female	29	1.1	No	No	Yes	No	No	Yes	Omnivore	Negative	-	-	-
S1-ZIM-03	Europe	Switzerland	Female	29	1	Yes	No	Yes	No	No	Yes	Omnivore	Negative	-	-	-
S1-BDI-01	Africa	Burundi	Male	34	0.8	Yes	No	No	Yes	No	Yes	Omnivore	Positive	3GC-R	CTX-M-15	ST5614
S1-BDI-02	Africa	Burundi	Female	23	0.4	Yes	No	Yes	Yes	No	No	Omnivore	Positive	3GC-R	SHV-5	ST429
S1-BUR-01	Africa	Burkina Faso	Female	30	1	Yes	No	Yes	Yes	No	Yes	Vegetarian	Negative	-	-	-
S1-EGY-01	Africa	Egypt	Male	54	1.3	Yes	No	No	No	No	No	Omnivore	Negative	-	-	-
S1-ETH-02	Africa	Ethiopia	Female	51	1.8	Yes	No	Yes	Yes	No	Yes	Omnivore	Positive	3GC-R	CTX-M-15	ST450
S1-ETH-03	Africa	Ethiopia	Female	40	2	Yes	Yes	Yes	No	No	Yes	Vegetarian	Positive	3GC-R	CTX-M-15	ST59
S1-ETH-04	Africa	Ethiopia	Female	64	1.1	Yes	No	No	No	No	Yes	Omnivore	Positive	3GC-R	CTX-M-3	ST394
S1-ETH-05	Africa	Ethiopia	Male	61	1.1	Yes	No	Yes	No	No	Yes	Omnivore	Positive	3GC-R	CTX-M-15	ST349
S1-ETH-06	Africa	Ethiopia	Male	53	2.5	No	No	No	Yes	No	Yes	Omnivore	Positive	3GC-R	CTX-M-15	ST13823
S1-ETH-07	Africa	Ethiopia	Female	52	2.5	Yes	No	No	Yes	No	Yes	NA	Negative	-	-	-
S1-GHA-01	Africa	Ghana	Female	32	0.4	No	No	Yes	Yes	No	Yes	Vegetarian	Negative	-	-	-
S1-IVC-01	Africa	Ivory Coast	Male	50	2.5	No	Yes	Yes	No	No	Yes	Omnivore	Negative	-	-	-
S1-IVC-02	Africa	Ivory Coast	Female	50	2.4	Yes	No	Yes	No	No	No	Omnivore	Positive	3GC-R	CTX-M-24	ST10

S1-KEN-01	Africa	Kenya	Male	26	0.7	Yes	No	No	No	No	Yes	Omnivore	Negative	-	-	-
S1-KEN-02	Africa	Kenya	Female	39	1.6	Yes	No	No	No	No	Yes	Omnivore	Negative	-	-	-
S1-KEN-03	Africa	Kenya	Female	40	0.4	No	No	Yes	No	No	Yes	Vegetarian	Positive	3GC-R	CTX-M-15	ST5614
S1-KEN-04	Africa	Kenya	Male	59	1.2	No	No	No	No	No	Yes	Omnivore	Positive	3GC-R	CTX-M-15	ST5614
S1-KEN-05	Africa	Kenya	Male	39	5	No	No	No	No	No	No	Omnivore	Positive	3GC-R	CTX-M-27	ST131
S1-KEN-06	Africa	Kenya	Female	31	30.2	No	No	No	No	No	No	Omnivore	Positive	3GC-R	CTX-M-15	ST450
S1-KEN-07	Africa	Kenya	Male	58	1.3	Yes	No	Yes	No	No	Yes	Omnivore	Positive	3GC-R	CTX-M-15	ST1193
S1-KEN-08	Africa	Kenya	Male	44	1.3	Yes	No	No	No	No	No	Omnivore	Negative	-	-	-
S1-KEN-09	Africa	Kenya	Female	31	1.5	Yes	No	Yes	No	No	Yes	Vegetarian	Positive	3GC-R	CTX-M-15	ST44
S1-KEN-10	Africa	Kenya	Male	33	1.3	Yes	No	Yes	No	No	Yes	Omnivore	Negative	-	-	-
S1-KEN-11	Africa	Kenya	Female	35	1.3	Yes	No	No	No	No	Yes	Omnivore	Negative	-	-	-
S1-KEN-12	Africa	Kenya	Female	51	NA	Yes	No	No	No	No	Yes	Omnivore	Negative	-	-	-
S1-KEN-13	Africa	Kenya	Male	52	2.9	Yes	No	No	No	No	No	Omnivore	Negative	-	-	-
S1-MOZ-01	Africa	Mozambique	Male	46	0.8	Yes	No	No	No	No	No	Omnivore	Negative	-	-	-
S1-NGR-01	Africa	Nigeria	Female	57	2.2	Yes	No	No	No	No	Yes	Omnivore	Negative	-	-	-
S1-NGR-02	Africa	Nigeria	Male	27	0.9	No	No	Yes	No	No	No	NA	Positive	3GC-R	CTX-M-15	ST2332
S1-RSA-01	Africa	South Africa	Male	60	0.7	Yes	No	No	No	No	No	Omnivore	Negative	-	-	-
S1-RSA-02	Africa	South Africa	Male	59	0.7	Yes	No	No	No	No	Yes	Vegetarian	Negative	-	-	-
S1-SEN-01	Africa	Senegal	Female	58	2.5	Yes	No	Yes	No	No	No	NA	Positive	3GC-R	CTX-M-15	ST219
S1-SUD-01	Africa	Sudan	Female	43	1	Yes	No	No	No	No	Yes	Omnivore	Positive	3GC-R	CTX-M-15	ST15576
S1-SUD-02	Africa	Sudan	Female	50	0.6	No	No	Yes	No	No	Yes	Vegetarian	Negative	-	-	-
S1-TAN-01	Africa	Tanzania	Female	55	2.2	Yes	No	No	No	No	Yes	Omnivore	Negative	-	-	-
S1-TAN-02	Africa	Tanzania	Male	54	2.3	Yes	No	No	No	No	No	Omnivore	Negative	-	-	-
S1-TAN-03	Africa	Tanzania	Male	34	0.8	Yes	No	No	No	No	No	Omnivore	Positive	Col-R	MCR-1.1	ST1196
S1-ZIM-01	Africa	Zimbabwe	Male	64	7.3	No	No	No	No	Yes	No	Omnivore	Positive	3GC-R	CTX-M-15	ST69
S1-ZIM-02	Africa	Zimbabwe	Female	58	0.4	Yes	No	Yes	No	No	No	Omnivore	Negative	-	-	-

Note. NA, not available; -, not applicable; 3GC-R, third-generation cephalosporin-resistant; Col-R, colistin-resistant; ST, sequence type; multidrug-resistant, MDR; Ent, Enterobacterales.

[a] Representative demographics and risk factors previously analyzed for the risk of intestinal colonization with MDR-Ent (Campos-Madueno *et al*., 2025; https://doi.org/10.1007/s10096-025-05069-w).

[b] Results of the culture-based intestinal colonization screening and phenotype of the colonizing strain as described in Campos-Madueno *et al*. (2025).

[c] Main resistance mechanism of the isolated strain associated with the observed phenotype and its sequence type as described in Campos-Madueno *et al*. (2025).

Table S2. Overall proportion of dereplicated MAG bins and their putative taxonomic lineage containing ARGs not present in plasmid contigs.

Europe MAGs (n=32) [a]					Africa MAGs (n=39)				
**Marker lineage [b]**	**Total bins**	**Unique bins [d]**	**Unique ARGs [f]**	**Bin prop (%) [h]**	**Marker lineage [c]**	**Total bins**	**Unique bins [e]**	**Unique ARGs [g]**	**Bin prop (%) [i]**
o__Clostridiales (UID1212)	52	37	23	36.6	o__Clostridiales (UID1212)	58	47	26	33.3
k__Bacteria (UID2372)	13	7	10	6.9	o__Clostridiales (UID1226)	22	17	12	12.1
o__Clostridiales (UID1226)	10	7	7	6.9	o__Bacteroidales (UID2657)	19	12	9	8.5
o__Bacteroidales (UID2657)	12	5	7	5.0	p__Actinobacteria (UID2112)	14	9	5	6.4
o__Lactobacillales (UID544)	9	5	4	5.0	f__Lachnospiraceae (UID1256)	7	6	5	4.3
o__Bacteroidales (UID2654)	8	4	6	4.0	f__Lachnospiraceae (UID1286)	9	6	6	4.3
f__Bifidobacteriaceae (UID1458)	7	3	3	3.0	g__Prevotella (UID2724)	7	6	3	4.3
f__Bifidobacteriaceae (UID1462)	4	3	3	3.0	k__Bacteria (UID2372)	7	6	5	4.3
g__Prevotella (UID2724)	3	3	1	3.0	f__Bifidobacteriaceae (UID1458)	8	5	5	3.5
o__Clostridiales (UID1120)	4	3	4	3.0	p__Bacteroidetes (UID2605)	4	4	3	2.8
f__Lachnospiraceae (UID1256)	3	2	3	2.0	k__Bacteria (UID203)	4	3	3	2.1
f__Lachnospiraceae (UID1286)	3	2	2	2.0	o__Bacteroidales (UID2654)	6	3	5	2.1
g__Bacteroides (UID2691)	10	2	9	2.0	p__Firmicutes (UID1022)	8	3	6	2.1
k__Bacteria (UID203)	2	2	2	2.0	f__Bifidobacteriaceae (UID1462)	4	2	2	1.4
k__Bacteria (UID2982)	2	2	1	2.0	c__Clostridia (UID1118)	2	1	2	0.7
o__Bacteroidales (UID2716)	4	2	4	2.0	f__Enterobacteriaceae (UID5124)	8	1	8	0.7
p__Actinobacteria (UID2112)	2	2	1	2.0	f__Enterobacteriaceae (UID5162)	3	1	3	0.7
p__Bacteroidetes (UID2605)	2	2	2	2.0	f__Lachnospiraceae (UID1255)	3	1	3	0.7
p__Firmicutes (UID1022)	4	2	3	2.0	g__Bacteroides (UID2691)	1	1	1	0.7
f__Enterobacteriaceae (UID5162)	2	1	2	1.0	g__Streptococcus (UID684)	2	1	2	0.7
k__Bacteria (UID2329)	2	1	2	1.0	k__Bacteria (UID2329)	1	1	1	0.7
o__Bacteroidales (UID2617)	1	1	1	1.0	k__Bacteria (UID2982)	2	1	1	0.7
o__Bacteroidales (UID2621)	5	1	5	1.0	o__Bacteroidales (UID2621)	2	1	1	0.7
o__Lactobacillales (UID374)	1	1	1	1.0	o__Clostridiales (UID1120)	1	1	1	0.7
o__Selenomonadales (UID1024)	3	1	1	1.0	o__Clostridiales (UID1375)	1	1	1	0.7
-	-	-	-	-	o__Lactobacillales (UID374)	1	1	1	0.7
**Total=**	168	101	107	~100	**Total=**	203	140	119	~100

Note. MAG, metagenome-assembled genome; ARGs, antimicrobial resistance genes. The top and other matching marker lineages of 'o_Clostridiales' are highlighted in green. See Supplementary Data 2 for complete dataset.

[a] No high-quality, dereplicated bins (bin-score >0.5) were determined from the S1-ESP-02 MAG, and so were excluded from this analysis.

[b, c] The specific marker lineage genes predicted by CheckM were used to classify each MAG bin.

[d, e] The number of unique bins out of the total number of bins per sample and per marker lineage containing ARGs.

[f, g, h, i] The number of unique ARGs in each sample and in each marker lineage, as well as the proportion of bins that match the corresponding lineage.

Table S3. Genus and species-level classification with GTDB-Tk of high-quality MAG bins containing ARGs not present in plasmid contigs.

Continent [a]	Genus [b]	Total bins [c]	Unique bins [d]	Bin prop. (%) [e]	Total species [f]	Unique species [g]	Species [h]	Total ARGs [i]	Unique ARGs [j]	List of unique ARGs [k]
Europe	g__Ruminococcoides	6	5	10.9	6	2	s__Ruminococcoides intestinale, s__Ruminococcoides sp002491825	6	4	erm(B)_AF299292, tet(32)_EF626943, tet(O)_Y07780, tet(W)_AJ427422
Europe	g__Bifidobacterium	9	4	8.7	9	3	s__Bifidobacterium adolescentis, s__Bifidobacterium bifidum, s__Bifidobacterium longum	9	4	erm(X)_U21300, erm(X)_X51472, tet(O)_M18896, tet(W)_FN396364
Europe	g__Bacteroides	6	3	6.5	6	1	s__Bacteroides uniformis	6	6	ant(6)-Ia_KF864551, cfxA_U38243, cfxA3_AF472622, cfxA4_AY769933, cfxA5_AY769934, tet(Q)_X58717
Europe	g__Akkermansia	2	2	4.3	2	1	s__Akkermansia muciniphila	2	1	lnu(C)_AY928180
Europe	g__Collinsella	2	2	4.3	2	2	s__Collinsella sp019041915, s__Collinsella sp902388545	2	1	tet(W)_FN396364
Europe	g__Coprococcus	2	2	4.3	2	2	s__Coprococcus ammoniilyticus, s__Coprococcus eutactus	2	2	erm(B)_X72021, tet(O)_M18896
Europe	g__Dialister	4	2	4.3	4	2	s__Dialister sp000434475, s__Dialister succinatiphilus	4	3	ant(6)-Ia_KF864551, blaACI-1_AJ007350, tet(W)_AJ427422
Europe	g__Enterococcus_B	4	2	4.3	4	1	s__Enterococcus_B lactis	4	2	aac(6')-Ii_L12710, msr(C)_AF313494
Europe	g__Eubacterium_R	4	2	4.3	4	2	s__Eubacterium_R sp000434995, s__Eubacterium_R sp000436835	4	4	aac(6')-aph(2")_M13771, erm(B)_U18931, erm(B)_X72021, tet(W)_AJ427422
Europe	g__Fusicatenibacter	5	2	4.3	5	1	s__Fusicatenibacter saccharivorans	5	5	ant(6)-Ia_KF864551, lnu(C)_AY928180, mef(A)_AF227520, msr(D)_AF227520, tet(32)_EF626943
Europe	g__Phocaeicola	3	2	4.3	3	2	s__Phocaeicola dorei, s__Phocaeicola vulgatus	3	3	cfxA3_AF472622, erm(B)_U18931, tet(Q)_L33696
Europe	g__Allocoprobacillus	1	1	2.2	1	1	s__Allocoprobacillus merdigallinarum	1	1	tet(44)_FN594949
Europe	g__Anaerobutyricum	3	1	2.2	3	1	s__Anaerobutyricum hallii	3	3	catS_X74948, erm(B)_X72021, tet(32)_EF626943
Europe	g__Anthropogastromicrobium	1	1	2.2	1	1	s__Anthropogastromicrobium aceti	1	1	ant(6)-Ia_KF864551
Europe	g__Bacteroides_F	1	1	2.2	1	1	s__Bacteroides_F pectinophilus	1	1	tet(O)_Y07780
Europe	g__Blautia_A	1	1	2.2	1	1	s__Blautia_A wexlerae	1	1	tet(O)_M18896
Europe	g__Butyrivibrio_A	1	1	2.2	1	1	s__Butyrivibrio_A crossota	1	1	tet(O)_Y07780
Europe	g__CAG-217	2	1	2.2	2	1	s__CAG-217 sp000436335	2	2	erm(B)_AF299292, lnu(C)_AY928180
Europe	g__Clostridium_AQ	2	1	2.2	2	1	s__Clostridium_AQ innocuum	2	2	tet(M)_AM990992, tet(M)_X75073
Europe	g__Escherichia	2	1	2.2	2	1	s__Escherichia coli	2	2	blaTEM-1B_AY458016, blaTEM-95_AJ308558
Europe	g__F23-B02	1	1	2.2	1	1	s__F23-B02 sp003533405	1	1	tet(32)_EF626943
Europe	g__Fusicatenibacter_A	3	1	2.2	3	1	s__Fusicatenibacter_A intestinipullorum	3	1	lnu(C)_AY928180
Europe	g__Hominenteromicrobium	2	1	2.2	2	1	s__Hominenteromicrobium mulieris	2	2	tet(40)_AM419751, tet(O/32/O)_AIOQ01000025

Europe	g__Lachnospira	1	1	2.2	1	1	s__Lachnospira eligens_A	1	1	tet(O)_Y07780
Europe	g__Parabacteroides	5	1	2.2	5	1	s__Parabacteroides distasonis	5	5	cfxA_U38243, cfxA3_AF472622, cfxA4_AY769933, cfxA5_AY769934, tet(Q)_L33696
Europe	g__Prevotella	1	1	2.2	1	1	s__Prevotella copri_K	1	1	cfxA6_GQ342996
Europe	g__Roseburia_C	2	1	2.2	2	1	s__Roseburia_C amylophila	2	2	tet(O)_M18896, tet(O)_M20925
Europe	g__Ruminiclostridium_E	2	1	2.2	2	1	s__Ruminiclostridium_E sp003512525	2	2	ant(6)-Ia_KF864551, tet(W)_AJ427422
Europe	g__UBA11524	1	1	2.2	1	1	s__UBA11524 sp000437595	1	1	tet(32)_EF626943
	**Total=**	79	46	~100	79	37	**Total=**	79	65	-
Africa	g__Ruminococcoides	13	9	19.6	13	2	s__Ruminococcoides intestinale, s__Ruminococcoides intestinalis	13	7	erm(G)_M15332, tet(M)_U58985, tet(O)_M20925, tet(O)_Y07780, tet(O/32/O)_AJ295238, tet(O/32/O)_NG_048124, tet(W)_AJ427422
Africa	g__Bacteroides	5	5	10.9	5	2	s__Bacteroides cellulosilyticus, s__Bacteroides uniformis	5	3	cfxA3_AF472622, erm(F)_M17808, tet(Q)_L33696
Africa	g__Agathobacter	3	2	4.3	3	1	s__Agathobacter rectalis	3	3	ant(6)-Ia_KF864551, tet(O)_M20925, tet(O/32/O)_AIOQ01000025
Africa	g__Anaerostipes	2	2	4.3	2	1	s__Anaerostipes hadrus	2	2	catS_X74948, tet(M)_EU182585
Africa	g__Anaerotardibacter	4	2	4.3	4	2	s__Anaerotardibacter sp000435475, s__Anaerotardibacter sp900556585	4	4	erm(B)_U18931, tet(W)_AJ427422, tet(W)_DQ060146, tet(W)_FN396364
Africa	g__Bifidobacterium	4	2	4.3	4	2	s__Bifidobacterium adolescentis, s__Bifidobacterium pseudocatenulatum	4	3	tet(W)_AJ427422, tet(W)_DQ060146, tet(W)_FN396364
Africa	g__Blautia_A	3	2	4.3	3	1	s__Blautia_A wexlerae	3	3	ant(6)-Ia_KF864551, tet(O)_M18896, tet(O)_M20925
Africa	g__Catenibacterium	3	2	4.3	3	2	s__Catenibacterium mitsuokai, s__Catenibacterium mitsuokai_A	3	2	erm(B)_U18931, tet(M)_X90939
Africa	g__Collinsella	2	2	4.3	NA	NA	NA	2	1	tet(W)_FN396364
Africa	g__Eubacterium_R	4	2	4.3	4	2	s__Eubacterium_R sp000436835, s__Eubacterium_R sp003526845	4	4	ant(6)-Ia_KF864551, tet(O/32/O)_NZ_AUJS01000017, tet(W)_AJ427422, tet(W)_FN396364
Africa	g__Prevotella	3	2	4.3	3	2	s__Prevotella copri_C, s__Prevotella sp015074785	3	2	cfxA6_GQ342996, tet(Q)_L33696
Africa	g__Akkermansia	2	1	2.2	2	1	s__Akkermansia muciniphila	2	1	lnu(C)_AY928180
Africa	g__Coprococcus	1	1	2.2	1	1	s__Coprococcus ammoniilyticus	1	1	tet(O)_Y07780
Africa	g__Cryptobacteroides	1	1	2.2	1	1	s__Cryptobacteroides sp000433355	1	1	tet(Q)_L33696
Africa	g__Dialister	4	1	2.2	4	1	s__Dialister sp002320515	4	3	blaACI-1_AJ007350, tet(W)_AJ427422, tet(W)_DQ060146
Africa	g__Eisenbergiella	3	1	2.2	3	1	s__Eisenbergiella porci	3	3	aac(6')-Im_AF337947, aph(2")-Ib_AF207840, tet(W)_AJ427422
Africa	g__Escherichia	8	1	2.2	8	1	s__Escherichia coli	8	8	aph(3")-Ib_AF321551, aph(6)-Id_M28829, blaCTX-M-15_AY044436, blaTEM-1B_AY458016, dfrA17_FJ460238, mph(A)_D16251, sul2_HQ840942, tet(B)_AF326777
Africa	g__Faecalimonas	4	1	2.2	4	1	s__Faecalimonas phoceensis	4	4	ant(6)-Ia_AF330699, ant(6)-Ia_KF864551, aph(3')-III_M26832, erm(B)_U18931
Africa	g__Fimenecus	1	1	2.2	1	1	s__Fimenecus sp004556705	1	1	tet(W)_AJ427422

Africa	g__Fusicatenibacter	1	1	2.2	1	1	s__Fusicatenibacter saccharivorans	1	1	ant(6)-Ia_KF864551
Africa	g__Holdemanella	1	1	2.2	1	1	s__Holdemanella sp900556915	1	1	tet(M)_U58985
Africa	g__Ligilactobacillus	1	1	2.2	1	1	s__Ligilactobacillus ruminis	1	1	tet(M)_FR671418
Africa	g__Mediterraneibacter	1	1	2.2	1	1	s__Mediterraneibacter faecis	1	1	tet(O/32/O)_AIOQ01000025
Africa	g__Ruminiclostridium_E	1	1	2.2	1	1	s__Ruminiclostridium_E siraeum	1	1	tet(32)_EF626943
Africa	g__UBA11524	1	1	2.2	1	1	s__UBA11524 sp000437595	1	1	catP_U15027
	**Total=**	76	46	~100	74	31	**Total=**	76	62	-

Note. Prop., proportion; '-', not applicable; '~', approximate.

[a] MAG bins corresponding to European and African MAGs are highlighted in green and orange, respectively.

[b, h] Taxonomic classification of MAGs with GTDB-Tk shown at the genus and species level. See Supplementary Data 2 for complete taxonomic rank classification.

[c, d, e] The total number of bins and the number of unique bins per sample and per genus, as well as the corresponding proportion based on unique bins.

[f, g, h] Total and unique number of species identified within a given bin, and corresponding list of unique species.

[i, j, k] The total and unique number of ARGs identified within the same bins (i.e., those classified at the genus and species level), as well as the corresponding list of ARGs.

Table S4. Summary of stool meta-assembled genomes (MAGs, n=28) associated with contigs containing antimicrobial resistance genes (ARGs) and plasmid replicon sequences.

Sample [a]	MAG characterization [b]				BLASTn (database: PLSDB) [c]		MASH screen (database: PLSDB) [d]	
	**Contig**	**Length (bp)**	**ARGs**	**Plasmid [replicon sequence(s)]**	**Top hit**	**Plasmid host**	**Top hit**	**Plasmid host**
S1-BEL-01	contig_827	85865	*aph(6)-Id, aph(3")-Ib, sul2*	IncB/O/K/Z [CU928147 (*Escherichia coli*)]	NZ_CP051702.1 (93339 bp; 100% cov, 99.95% id)	*Escherichia coli*	CP101345.1 (2640 bp; 977 sh)	*Salmonella enterica*
S1-CHI-01	contig_1402	84548	*tet(M)*	repUS43 [CP003584 (*Enterococcus faecium*)]	Below threshold	NA	NZ_KP345886.1 (23910 bp; 480 sh)	*Enterococcus faecium*
S1-DNK-01	contig_16	467999	*aac(6')-aph(2"), erm(B)*	*rep1* [AE016833 (*Enterococcus faecalis*) [	Below threshold	NA	NZ_CP151258.1 (1918 bp; 996 sh)	*Staphylococcus aureus*
S1-ESP-02	contig_97	13267	*aac(6')-aph(2"), erm(B)*	rep1 [AE016833 (*Enterococcus faecalis*)]	NZ_CP118266.1 (166452 bp; 96% cov, 99.76% id)	*Clostridium perfringens*	NZ_CP151258.1 (1918 bp; 996 sh)	*Staphylococcus aureus*
S1-ESP-03	contig_5088	72409	$\mathit{bla}_{\text{CTX-M-15}}$, *qnrS1*	IncFII [AY458016 (*Escherichia coli*)]	NZ_CP145621.1 (72411 bp; 100% cov, 100% id) *	*Escherichia coli*	NZ_CP079817.1 (1657 bp; 1000 sh)	*Klebsiella pneumoniae*
S1-GBR-01	contig_9715	14724	*aph(6)-Id*	IncQ1 [M28829 (*Escherichia coli*)]	NZ_CP030774.1 (10866 bp; 42% cov, 99.88% id)	*Shigella flexneri 1c*	NZ_CP142411.1 (6477 bp; 747 sh)	*Klebsiella pneumoniae*
S1-GER-04	contig_3069	15996	*tet(M)*	repUS43 [CP003584 (*Enterococcus faecium*)]	LR962312.1 (70709 bp; 88% cov, 99.86% id)	*Enterococcus faecalis*	NZ_KP345886.1 (23910 bp; 474 sh)	*Enterococcus faecium*
S1-KOS-02	contig_1136	29633	*aadA2, mph(A), sul1, tet(A), dfrA12*	IncFII(29) [CP003035 (*Escherichia coli*)]	CP119492.1 (222461 bp; 68% cov, 99.86% id)	*Salmonella enterica* subsp. *enterica* serovar Schwarzengrund	NZ_CP084050.1 (3161 bp; 1000 sh)	*Klebsiella pneumoniae*
S1-POR-01	contig_5648	5754	*erm(B)*	repUS47 [FJ374272 (*Lactobacillus plantarum*)]	NC_020237.1 (11951 bp; 77% cov, 99.43% id)	*Staphylococcus hyicus*	NC_012628.1 (4031 bp; 954 sh)	*Lactiplantibacillus plantarum*
S1-SRB-01	contig_9013	74196	$\mathit{bla}_{\text{TEM-1D}}$	IncFII [AY458016 (*Escherichia coli*)]	NZ_AP022288.1 (127169 bp; 72% cov, 97.36% id)	*Escherichia coli*	NZ_CP083488.1 (3780 bp; 745 sh)	*Escherichia coli*
S1-SRB-02	contig_1077	88405	*aac(6')-aph(2"), erm(B)*	rep1 [AE016833 (*Enterococcus faecalis*)]	Below threshold	NA	NZ_CP151258.1 (1918 bp; 931 sh)	*Staphylococcus aureus*
S1-BDI-01	contig_6335	77066	$\mathit{bla}_{\text{TEM-1B}}$	IncFII(29) [CP003035 *Escherichia coli*)]	NZ_CP049848.1 (68488 bp; 71% cov, 97.60% id)	*Escherichia coli*	NZ_CP079817.1 (1657 bp; 1000 sh)	*Klebsiella pneumoniae*
	contig_2191	16593	*aph(6)-Id, aph(3")-Ib, sul2, tet(B)*	IncQ1 [M28829 (*Escherichia coli*)]	NZ_CP116195.1 (108065 bp; 72% cov, 99.73% id)	*Escherichia coli*	CP101346.1 (1746 bp; 964 sh)	*Salmonella enterica*
S1-BDI-02	contig_57	224305	*tet(M)*	repUS43 [CP003584 (*Enterococcus faecium*)]	Below threshold	NA	NZ_KP345886.1 (23910 bp; 541 sh)	*Enterococcus faecium*
S1-BUR-01	contig_2326	198075	*tet(M)*	repUS43 [CP003584 (*Enterococcus faecium*)]	Below threshold	NA	NZ_KP345886.1 (23910 bp; 528 sh)	*Enterococcus faecium*
S1-ETH-02	contig_236	65256	*tet(M)*	repUS43 [CP003584 (*Enterococcus faecium*)]	Below threshold	NA	NZ_KP345886.1 (23910 bp; 540 sh)	*Enterococcus faecium*
S1-ETH-04	contig_1599	180095	*aac(6')-aph(2"), erm(B)*	rep1 [AE016833 (*Enterococcus faecalis*)]	Below threshold	NA	NZ_CP151258.1 (1918 bp; 1000 sh)	*Staphylococcus aureus*
	contig_5505	34147	*tet(M)*	repUS43 [CP003584 (*Enterococcus faecium*)]	NZ_AP026824.1 (112887 bp; 52% cov, 99.45% id)	*Clostridium tetani*	NZ_KP345886.1 (23910 bp; 528 sh)	*Enterococcus faecium*
S1-ETH-05	contig_1957	23417	*tet(M)*	repUS43 [CP003584 (*Enterococcus faecium*)]	LR962405.1 (70355 bp; 72% cov, 99.59% id)	*Enterococcus faecalis*	NZ_KP345886.1 (23910 bp; 542 sh)	*Enterococcus faecium*
S1-IVC-01	contig_1871	120205	*tet(M)*	repUS43 [CP003584 (*Enterococcus faecium*)]	Below threshold	NA	NZ_KP345886.1 (23910 bp; 541 sh)	*Enterococcus faecium*
	contig_1357	34663	*tet(B)*	IncFIB(AP001918) [AP001918 (*Escherichia coli*)]	NZ_CP020496.1 (120668 bp; 89% cov, 99.98% id)	*Escherichia coli*	NZ_CP149265.1 (19583 bp; 717 sh)	*Salmonella enterica*
S1-KEN-02	contig_1160	169555	*lnu(C), tet(M)*	repUS43 [CP003584 (*Enterococcus faecium*)]	Below threshold	NA	NZ_KP345886.1 (23910 bp; 549 sh)	*Enterococcus faecium*
S1-KEN-03	contig_103	14288	*aac(6')-aph(2"), erm(B)*	rep1 [AE016833 (*Enterococcus faecalis*)]	NZ_CP118266.1 (166452 bp; 92% cov, 99.80% id)	*Clostridium perfringens*	NZ_CP151258.1 (1918 bp; 996 sh)	*Staphylococcus aureus*
	contig_1335	116950	*tet(M)*	repUS43 [CP003584 (*Enterococcus faecium*)]	Below threshold	NA	NZ_KP345886.1 (23910 bp; 522 sh)	*Enterococcus faecium*
S1-KEN-05	contig_530	136055	*tet(M)*	repUS43 [CP003584 (*Enterococcus faecium*)]	Below threshold	NA	NZ_KP345886.1 (23910 bp; 407 sh)	*Enterococcus faecium*

S1-KEN-07	contig_2849	102328	*aph(6)-Id, aph(3'')-Ib, bla*$_{TEM-1B}$, *mph(A), sul2, tet(B), dfrA17*	Col156 [NC009781 (*Escherichia coli*)], IncFIA/FIB(AP001918) [AP001918 (*Escherichia coli*)], IncQ1 [M28829 (*Escherichia coli*)]	NZ_CP145684.1 (102327 bp; 100% cov, 100% id) *	*Escherichia coli*	NZ_CP084054.1 (1355 bp; 405 sh)	*Klebsiella pneumoniae*
S1-KEN-11	contig_3204	147602	*aph(6)-Id, aph(3'')-Ib, aadA5, bla*$_{TEM-1B}$, *mph(A), sul1, sul2, tet(B), dfrA17*	Col156 [NC009781 (*Escherichia coli*)], IncFIB(AP001918) [AP001918 (*Escherichia coli*)], IncFII(29) [CP003035 (*Escherichia coli*)], IncQ1 [M28829 (*Escherichia coli*)]	NZ_CP146688.1 (134947 bp; 92% cov, 100% id)	*Escherichia coli*	NC_025014.1 (2154 bp; 557 sh)	*Escherichia coli*
S1-KEN-12	contig_7056	29703	*tet(A)*	IncFII(pRSB107) [AJ851089 (Uncultured bacterium)]	NZ_CP042972.1 (129967 bp; 66% cov, 99.54% id)	*Escherichia coli*	CP051409.1 (9083 bp; 1000 sh)	*Salmonella enterica*
S1-KOS-01	contig_2043	122895	*tet(M)*	repUS43 [CP003584 (*Enterococcus faecium*)]	Below threshold	NA	NZ_KP345886.1 (23910 bp; 527 sh)	*Enterococcus faecium*
S1-NGR-02	contig_510	112468	*bla*$_{CTX-M-15}$	IncFIB(H89-PhagePlasmid) [HG530657 (*Escherichia coli*)]	NZ_CP145681.1 (113079 bp; 100% cov, 100% id) *	*Escherichia coli*	NZ_CP079130.1 (1449 bp; 1000 sh), NZ_CP145681.1 (113079 bp; 984 sh)	*Klebsiella pneumoniae, Escherichia coli*
	contig_67	95723	*bla*$_{TEM-1B}$, *sul1, tet(A), dfrA7*	IncFIB(AP001918) [AP001918 (*Escherichia coli*)], IncFII(pHN7A8) [JN232517 (*Escherichia coli*)]	NZ_CP145680.1 (113912 bp; 100% cov, 99.97% id) *	*Escherichia coli*	NZ_CP079814.1, NZ_CP079817.1 (2710 bp, 1657 bp; 1000 sh (both)	*Klebsiella pneumoniae* (both)
S1-RSA-01	contig_644	155803	*tet(M)*	repUS43 [CP003584 (*Enterococcus faecium*)]	Below threshold	NA	NZ_KP345886.1 (23910 bp; 540 sh)	*Enterococcus faecium*
S1-SUD-01	contig_50	461036	*tet(M)*	repUS43 [CP003584 (*Enterococcus faecium*)]	Below threshold	NA	NZ_KP345886.1 (23910 bp; 546 sh)	*Enterococcus faecium*

Note. cov, coverage; id, identity; sh, shared hashes; NA, not applicable.

[a] Samples highlighted in green correspond to European stools, while those in orange to African stools.

[b] 'Contig' corresponds to the MAG header/identifier; contig length is given in base-pairs (bp). ARGs and plasmid replicon sequence(s) [replicon sequence name (underlined), plasmid accession number, plasmid host] are located in the same MAG contig.

[c] Nucleotide (n) BLAST results against the PLSDB database are shown here. Only the top hit (sorted by bit score and e-value) is shown: plasmid accession number (length; query coverage, identity) and plasmid host. Results with query coverage <40% (below threshold) were excluded. Results marked with an asterisk (*) correspond to plasmid hits previously confirmed by strain whole-genome sequencing (Campos-Madueno *et al.*, 2025; https://doi.org/10.1007/s10096-025-05069-w).

[d] The PLSDB MASH screening results are shown here. The result(s) with the most shared hashes (out of 1000) is shown: plasmid accession number (length; shared hashes) and plasmid host.
